# DrugDL: dual-modal deep learning framework for multi-property drug prediction and targeted therapy discovery

**DOI:** 10.1093/bioinformatics/btag392

**Published:** 2026-06-15

**Authors:** Qi Zhang, Xuan Yu, Yuxiao Wei, Yunpeng Xia, Long-Chen Shen, Zhi-Hui Wang, Hong-Bin Shen, Dong-Jun Yu

**Affiliations:** School of Computer Science and Engineering, Nanjing University of Science and Technology, Nanjing, 210094, China; Department of Computer Science, City University of Hong Kong, Kowloon Tong, Hong Kong, 999077, China; School of Computer Science and Technology, Beijing Jiaotong University, Beijing, 100044, China; Institute of Image Processing and Pattern Recognition, Shanghai Jiao Tong University, Shanghai, 200240, China; School of Computer Science and Engineering, Nanjing University of Science and Technology, Nanjing, 210094, China; School of Computer Science and Engineering, Nanjing University of Science and Technology, Nanjing, 210094, China; Institute of Image Processing and Pattern Recognition, Shanghai Jiao Tong University, Shanghai, 200240, China; School of Computer Science and Engineering, Nanjing University of Science and Technology, Nanjing, 210094, China

## Abstract

**Motivation:**

The accurate and robust representation of drug molecule features, the prediction of drug-target biomacromolecule interactions, and the determination of physicochemical properties are crucial in drug development. However, these tasks remain challenging due to issues such as the limited generalizability of single-modal representations, the absence of multitask prediction frameworks, and weak adaptability in cold-start scenarios.

**Results:**

In this study, we present DrugDL, a framework for comprehensive drug molecule representation and the prediction of multiple downstream tasks, including drug-target interactions, binding affinities, binding sites, physicochemical properties, toxicity, and drug–drug interactions. DrugDL jointly learns representations of the drug chemical space and the target protein biological space, while capturing multiscale interaction mechanisms between drug molecules and target proteins through the integration of cross-modal contrastive learning and single-modal feature enhancement algorithms. Specifically, DrugDL employs a multitask prediction framework to predict multiple properties of drug molecules. In practical applications, it consistently outperforms state-of-the-art methods, particularly in cold-start tasks. The framework has been successfully applied to high-throughput screening, the identification of inhibitors of SARS-CoV-2 and metabolic enzymes, and the prediction of cancer-targeted drugs. Experimental validations on EGFR and ALK targets further demonstrate its effectiveness as a precise drug discovery tool. By enabling accurate molecular representation and multi-property prediction, DrugDL provides end-to-end technical support for drug development, thereby significantly accelerating the drug discovery process.

**Availability and implementation:**

The datasets and code are available at https://github.com/ZhangQi9910/DrugDL. The version of record is archived in Zenodo with the DOI: 10.5281/zenodo.20579718.

## 1 Introduction

As a result of the rapid advancements in biomedicine, drug research and development (R&D) plays a pivotal role in enhancing human health and driving industrial innovation (Singh *et al.* 2023). At its core, this process involves decoding the interactions between drug molecules and biomacromolecules such as proteins and DNA, as well as understanding the physicochemical properties of drug molecules (Chen *et al.* 2016). These factors, including interaction patterns, target binding modes, and toxicity, determine key drug parameters, thereby forming the basis for screening and optimization. While experimental approaches remain essential in drug discovery, large-scale biochemical assays for interaction identification and property characterization are costly and time-consuming (DiMasi *et al.* 2003). Consequently, computational methods have been widely adopted, yielding notable progress in drug property prediction, shortening the R&D cycle and reducing costs (Zhu *et al.* 2023).

The core of computational methods for drug property prediction lies in identifying more efficient and rational approaches for representing the features of drugs (and target biomolecules) and in developing more accurate predictors (Li *et al.* 2024). With the rapid development of computer technology and deep learning, revolutionary changes have occurred in molecular feature representation methods (Boulougouri *et al.* 2024). Existing representation methods can be broadly categorized into three types: text-based representation (Weininger 1988), fingerprint-based representation (Honda *et al.* 2019), and graph-based representation (Zhang *et al.* 2025). Text-based representations encode drug molecule structural information as text to capture basic features. There are diverse text-based representation methods for drug molecules, among which the simplified molecular-input line-entry system (SMILES) representation and InChI representation (Handsel *et al.* 2021) are the most widely used. SMILES is a standardized language that uses short strings to represent chemical structures, offering simplicity, uniqueness, and reversibility, and serves as input for most drug property prediction models. Fingerprint-based representations convert chemical structure information into a 1D binary vector, where each bit indicates the presence or absence of a specific structural feature. Common molecular fingerprints include MACCS (Morgan 1965) and ECFP (Muegge and Mukherjee 2016). Graph-based molecular representations have attracted growing attention in recent years and are commonly categorized into molecular graphs (Fang *et al.* 2022) and molecular images (Zeng *et al.* 2022). These methods often rely on specific software or libraries, such as RDKit, which can parse SMILES strings, identify atomic and chemical bond information, construct molecular graphs, and generate images accordingly. A molecular graph is an abstract representation based on nodes and edges, focusing on the molecular skeleton and the connectivity of atoms (Axelrod and Gómez-Bombarelli 2022). With growing research on molecular substructures and functional groups, drug motif graphs have gradually emerged in many drug property prediction models (Yu and Gao 2022). Unlike drug atom graphs, drug motif graphs are higher-level graph models that focus on the structural fragments and their interactions. Molecular images are 2D graphical representations, typically constructed using red, green, and blue color channels and provide an intuitive and concise representation of molecular structures (Zhong *et al.* 2021).

In response to complex and diverse drug molecule property prediction tasks, researchers have developed various computational models tailored to different problems. In the prediction of drug-target interactions (DTIs), which are crucial in drug R&D, most methods use the linear and 2D structural information of drugs and targets as inputs (Ru *et al.* 2025), treat DTI prediction as a binary classification task, and employ different deep encoding and decoding modules, such as deep neural networks, graph neural networks (GNNs), convolutional neural networks (CNNs), and transformers, for prediction (Liu *et al.* 2023). Examples of such models include DrugBAN (Bai *et al.* 2023), ZeroBind (Wang *et al.* 2023), PSICHIC (Koh *et al.* 2024), DTIAM (Lu *et al.* 2025), and GraphBAN (Hadipour *et al.* 2025). With the rapid increase in the number of known target structures and the improvement in the accuracy of AlphaFold-like protein 3D structure prediction methods, a feasible approach for effectively modeling 3D target structures has emerged. Recently, many deep learning models have integrated 3D structure information to improve DTI prediction (Lim *et al.* 2019). To further predict the assumed strength of interactions in more detail, various regression-based models have been proposed to infer the binding affinities between drugs and targets (Hu *et al.* 2025). Binding affinity reflects the degree of binding between a drug and a specific target and can be quantified by indicators such as the inhibition constant $K_{i}$, dissociation constant $K_{d}$, and half-maximal inhibitory concentration ${IC}_{50}$. Models used to predict drug-target binding affinity (DTA) and DTI usually employ similar feature representation and extraction strategies, modifying only the final output mapping results and objective functions in the predictors. Examples of such models include KDBNet (Luo *et al.* 2023) and MMD-DTA (Zhang *et al.* 2024). Although these methods can successfully predict more detailed affinity strengths than DTI-type tasks, their ability to reveal more precise specific binding sites remains very limited. Therefore, many models introduce the attention mechanism to assign weights to features representing different sequence segments according to their importance levels to display specific binding sites. For example, Li *et al.* (2020) developed MONN, which uses noncovalent interactions as additional supervision information to guide the model to capture key binding sites. Hua *et al.* (2023) developed a multifunctional model, MFR-DTA, which employs a Mix-Decoder module and fully connected layers to treat the prediction of drug-target binding regions (DTBRs) as a supervised learning task.

In predicting physicochemical properties, toxicity, and drug–drug interactions (DDIs)—tasks directly related to drug safety—most models focus primarily on molecular structures. Unlike DTI and DTA tasks, which involve complex multimodal interactions, these models emphasize functional groups and local action regions of drug molecules (Yang *et al.* 2022). These methods can reveal substructures in drug molecules in two ways: explicitly and implicitly. Explicit models decompose drug molecules according to certain settings and then apply a tokenization process, similar to that used in natural language processing, for representation (Deng *et al.* 2022). In contrast, implicit models usually take molecular graphs as inputs and adopt a combination of a GNN and attention mechanisms to automatically extract substructures from each molecular graph (Xu *et al.* 2019). For example, the MolCLR model proposed by Wang *et al.* (2022) uses a GNN, conducts self-supervised learning with large-scale unlabeled data, and performs molecular property predictions in tasks such as predicting human β-secretase inhibitors (BACE), blood–brain barrier permeability (BBBP), and drug molecule toxicity while simultaneously identifying important substructures. Zeng *et al.* (2022) used image representations of drug molecules to detect various physicochemical properties and split the molecular structure by identifying clusters using a multigranular chemical cluster classification method. The MeTDDI method proposed by Zhong *et al.* (2024) predicts DDIs based on local-global self-attention and co-attention mechanisms, providing accurate explanations of the structural mechanisms of DDIs.

Despite extensive efforts in molecular representation and property prediction, current approaches still face notable limitations. First, most representation methods focus on single-modal drug molecule information, restricting their ability to capture deep-level features, especially those related to other biomolecules (Pourmousa *et al.* 2025). This limitation leads to poor generalizability in predicting new drug-target interactions, similar to the cold-start problem. In addition, many state-of-the-art drug molecule property prediction methods are often limited to one-sided predictions. For example, some such methods are designed solely for predicting DTI and DTA, whereas others can only be applied to DDI prediction. Comprehensive methods that can cover multiple important steps in the drug R&D process remain lacking. Moreover, previous methods also rely heavily on large-scale, high-quality labeled data and interconnected biomedical entities (diseases, side effects, etc.) (Song *et al.* 2024), which are scarce in early-stage drug discovery. More importantly, previous methods have failed to elucidate drug action mechanisms and have struggled to translate these insights into real-world virtual drug screening, remaining mainly theoretical, with limited practical validation of candidate drugs. Therefore, developing advanced deep learning models for multi-property prediction, mechanism exploration, and real-world application represents a critical yet highly challenging task in drug development.

**Figure 1:**
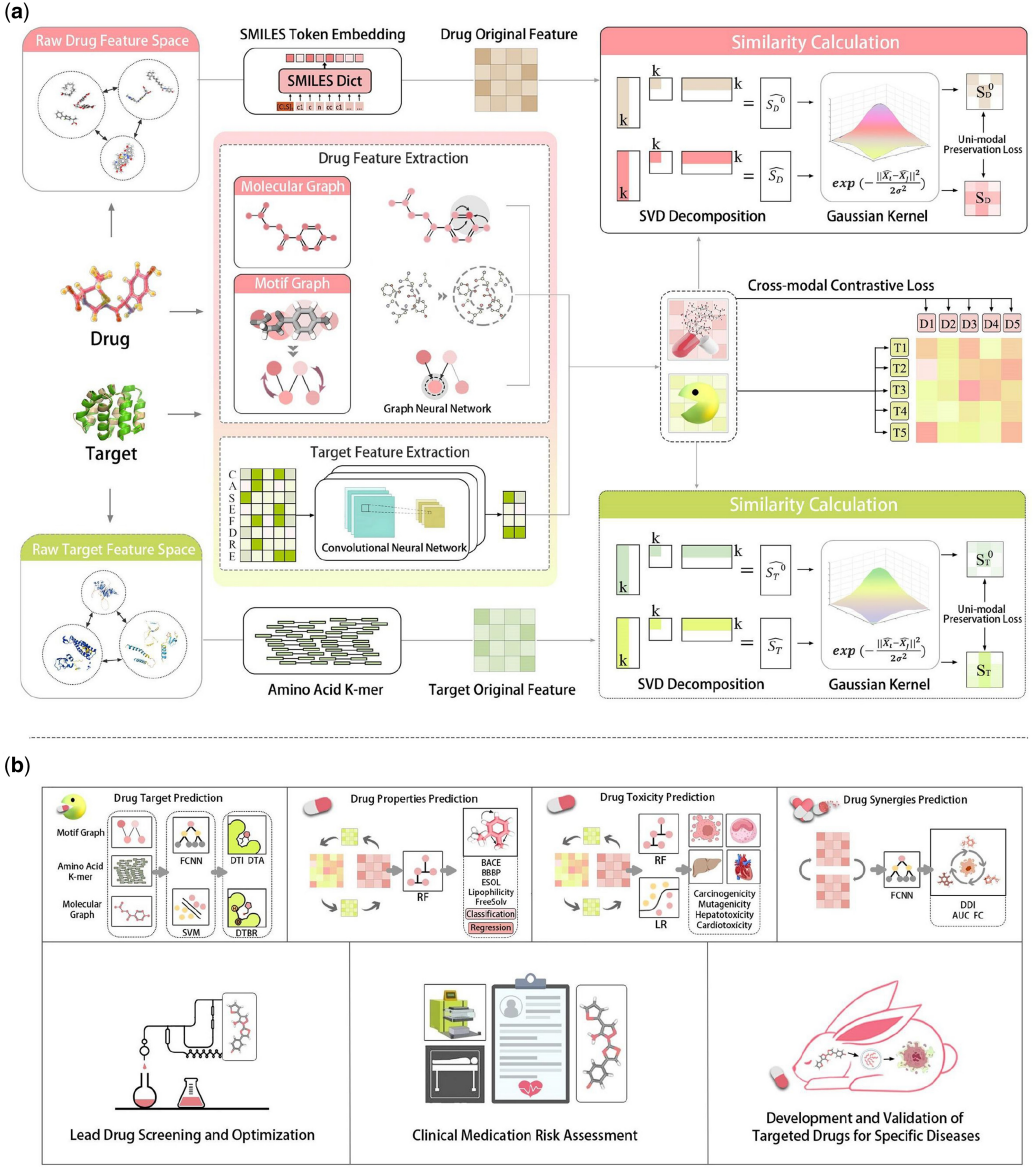
Architectural overview of DrugDL. (a) The framework consists of two main modules. Cross-modal interaction learning module: This module converts drug molecules into atomic-level topological and motif graphs, and maps target protein sequences into feature matrices. Multichannel GNNs and multilayer CNN blocks are used to learn drug and target embeddings. Integrates embeddings and aligns heterogeneous features into a unified space via cross-modal interaction learning. Single-modal feature enhancement module: This module calculates single-modal data similarity with matrix SVD and Gaussian kernel functions. A preservation loss is introduced to strengthen the internal feature relations within each modality. The two modules are jointly optimized for expressive features. (b) Schematic diagram of DrugDL application. The DrugDL model integrates multiple drug and target features and uses cross-modal interaction learning and single-modal feature enhancement techniques to facilitate training and testing on benchmark datasets. It can be applied to various downstream tasks, including the prediction of DTI, DTA, and DTBR and the prediction of molecular properties such as human β-secretase inhibitor (BACE) and blood–brain barrier permeability (BBBP). Moreover, DrugDL can predict toxicities, such as molecular carcinogenicity, mutagenicity, hepatotoxicity (DILI), and hERG cardiotoxicity, as well as DDI. Through decision analysis and interpretability analysis, the model can help elucidate drug mechanisms of action, provide suggestions for lead optimization, and evaluate clinical toxicity risks of drug molecules. DrugDL can also perform virtual screening of drug molecules for specific targets and verify the effectiveness and safety of the screened drug molecules on the basis of experiments, thereby supporting the development and verification of therapeutic drugs for specific diseases.

In this study, we developed DrugDL, a cross-modal deep learning architecture for drug molecule representation and multi-property prediction (Fig. 1). DrugDL takes the SMILES sequences of drug molecules and the amino acid sequences of targets as inputs, and constructs drug molecular graphs and motif graphs based on specific rules to represent molecular features at different granularities. It introduces cross-modal contrastive learning and single-modal feature enhancement strategies, using contrastive learning loss to align the features of drug and target modalities, while employing matrix singular value decomposition (SVD) and Gaussian kernel functions to compute intramodal data similarity and preserve internal modal relationships. This architecture enables multiscale analysis of drug-target interactions and joint representation of drug chemical and target biological spaces. Furthermore, it adopts a multitask prediction framework to evaluate multiple downstream tasks, including DTI, DTA, DTBR, drug physicochemical property, toxicity, and DDI prediction. In comprehensive comparison tests of various tasks and multiple scenarios (warm start and cold-start), DrugDL consistently outperformed state-of-the-art methods. In addition, we successfully identified effective inhibitors against SARS-CoV-2 and metabolic enzymes from a high-throughput molecular library, and provided theoretical support for drugs at different clinical trial stages by predicting drugs that interact with cancer targets. Virtual screening of candidate drugs for EGFR and ALK targets further confirmed that DrugDL can serve as an efficient and accurate drug discovery tool to facilitate drug R&D. These results indicate that DrugDL can accurately represent drug molecules and effectively predict their multiple properties, thus substantially advancing the drug discovery process.

## 2 Methods

### 2.1 Benchmark datasets

To comprehensively evaluate model performance, we integrated multiple authoritative public datasets to construct a multitask benchmark system encompassing core tasks such as DTI, DTA and DTBR prediction; physicochemical properties prediction; toxicity assessment; and DDI prediction. For the DTI task, we use the BindingDB (Liu *et al.* 2007), BioSNAP (Zitnik *et al.* 2018), and Human datasets (Liu *et al.* 2015). Among them, BindingDB contains more than 50 000 verified interaction data of small molecules and proteins. BioSNAP, derived from DrugBank, includes nearly 30 000 drug molecule–protein interactions. The Human dataset focuses on the prediction of human-specific interactions. For binding affinity prediction, we use the Davis (Davis *et al.* 2011) and KIBA (Tang *et al.* 2014) benchmark datasets. Davis provides dissociation constant data of the kinase family, and KIBA integrates multisource biological activity indicators and standardizes the scores. For binding site prediction, we use the sc-PDB dataset, which contains 16 000 experimentally verified protein–ligand binding sites (Kellenberger *et al.* 2006).

For physicochemical properties prediction, we integrated five subsets of MoleculeNet (Wu *et al.* 2018): human β-secretase inhibitor (BACE), blood–brain barrier permeability (BBBP), Estimated Solubility (ESOL), Lipophilicity, and Free Solvation (FreeSolv), covering nearly 10 000 drug molecules in total. Toxicity assessment includes four tasks: carcinogenicity, mutagenicity, human ether-à-go-go-related gene (hERG) cardiotoxicity, and drug-induced liver injury (DILI). The carcinogenicity and mutagenicity data integrate the CPDB (Gold *et al.* 2005), CCRIS (Cameron *et al.* 1988), and ISSCAN (Benigni *et al.* 2008) databases, covering nearly 10 000 molecules. The hERG toxicity dataset was constructed based on ChEMBL (Gaulton *et al.* 2017), PubChem (Kim *et al.* 2016), and BindingDB (Liu *et al.* 2007), comprising 20 000 molecules, with toxicity labels divided on the basis of whether the IC50 value is lower than 10 μM. The liver injury data integrates the LTKB, LiverTox, and Hepatox databases (Chen *et al.* 2011), covering clinical adverse reaction records of nearly 10 000 drugs.

For DDI prediction, we used DrugBank 5.1.8 (Wishart *et al.* 2018) to construct a four-class classification task and a regression task. The classification task defines four classes representing drug metabolism inhibition and enhancement relationships, and the regression task quantifies pharmacokinetic effects through area under the plasma concentration-time curve fold-change (AUC FC) values. All datasets were subjected to deduplication, arbitration of conflicting labels, and prioritization of experimental verification to ensure the quality and reproducibility of the data.

Detailed experimental settings, including data preprocessing steps, sample balancing strategies, and specific dataset splitting methods, are comprehensively described in Supplementary Note 3.

### 2.2 Cross-modal interaction learning module

For each drug molecule, we construct two types of graphs: a drug atom graph, in which atoms represent nodes and chemical bonds represent edges, and a drug motif graph, in which motifs represent nodes and edges are constructed between nodes on the basis of multiple principles. These two graphs reveal the structural features and interactions of drug molecules from different perspectives. The atom graph $G_{a}=(V_{a},E_{a})$, where $V_{a}$ represents the set of atom nodes and $E_{a}$ represents the set of edges composed of chemical bonds between atoms. A drug molecule contains n atoms, and each atom *i* (*i = 1,2,…,n*) corresponds to a node $v_{i}\in V_{a}$ in the graph. Each node in the atom graph precisely reflects the local structural features of the atom. The motif graph $G_{m}=(V_{m},E_{m})$, where $V_{m}$ represents the set of nodes composed of motifs and $E_{m}$ represents the set of edges between nodes. The key to constructing a drug motif graph lies in the motif extraction of drug molecules and the construction of edges between nodes. First, we split each drug molecule into multiple molecular fragments. Specifically, substructure fragments are broadly divided into three types: ring structures, noncyclic parts, and carbon–carbon single bonds. This substructure splitting method covers all drugs included in our benchmark dataset and ensures that the motif splitting of each drug is unique and deterministic. Then, under the guidance of domain knowledge (Ji *et al.* 2022), we construct edges connecting nodes (motifs) according to multiple principles. First, when two motifs share atoms, we add an edge to connect the two motif nodes. Second, if two motifs do not share atoms, we consider whether any atoms of one motif are adjacent to atoms of the other motif. If so, we add an edge to connect the two motif nodes; otherwise, they are not connected. In this way, we successfully construct the drug motif graph. With respect to the initial features of each motif node in the graph, we set them as the concatenation of all the atom features it contains.

To extract deeper information from drug molecules, we use multichannel GNNs to learn drug representations. Specifically, in addition to using two parallel GATs to extract features from the drug atom graph and motif graph, we also use a GCN with shared weights to extract the consistent features of the two graphs. First, given the atom graph $G_{a}=(V_{a},E_{a})$ as input, for each atom node $v_{i}$, we calculate the attention coefficient $\alpha_{ij}$ between it and its neighbor node $v_{j}$ as follows:


(1) αijk= exp ⁡(LR(eij))∑t≠i exp ⁡(LR(eit))

(2) eij=akT[Wkhi||Wkhj]

where $e_{ij}$ represents the attention weight between adjacent nodes, $a_{k}^{T}$ and $W_{k}$ are the trainable parameters of the k-th attention mechanism, and *‖* is the concatenation operation. *LR* represents the LeakyReLU activation function. $h_{i}$ and $h_{j}$ represent the feature representations of node $v_{i}$ and node $v_{j}$ in the drug atom graph, respectively, and *k* represents the number of attention heads in the GAT. After we obtain the *k*-th attention weight $\alpha_{ij}^{k}$ between node $v_{i}$ and node $v_{j}$, we use the following equation to aggregate the features of neighbor nodes, complete node updates, and obtain the features $H_{a}$ of each drug atom graph:


(3) Ha=BN[∑i=1nσ(1K∑k=1K∑j≠iαijkWkhj)]

where σ represents the activation function and *BN(·)* represents the batch normalization layer. Similarly, taking the drug motif graph $G_{m}=(V_{m},E_{m})$ as input, after multilayer GAT feature extraction, we obtain the feature representation matrix $H_{m}$ of each drug motif graph. In addition, to promote knowledge sharing among different graph structures, deepen understanding of various graphs, and ensure that the features extracted from the atom graph and the motif graph are semantically related, we develop a GCN model with shared weights to extract the consistent feature representations of the two graph structures. Given the feature representation matrix $H_{a}$ of the drug atom graph and $H_{m}$ of the motif graph as inputs, we use the following equations to aggregate the features of neighboring nodes, complete node updates, and obtain the feature representations $H_{a}^{c}$ and $H_{m}^{c}$ of each drug atom graph and motif graph in the common space:


(4) Hac=BN[∑i=1nσ(∑j∈Ni(1ciWchjnew+b))]

(5) Hmc=BN[∑i=1mσ(∑j∈N¯i(1c¯iWc¯h¯jnew+b¯))]

where $W_{c}$, $W_{\bar{c}}$, b, and $\bar{b}$ represent the trainable parameter matrices and biases in the GCN, respectively, and $c_{i}$ and ${\bar{c}}_{i}$ are normalization constants. Finally, we compute the average of the feature representations of the two graphs in the common space to obtain the final common feature representation $H_{am}$ of the drug molecule.

In the process of target feature representation learning, to comprehensively and accurately extract adjacent information in the protein sequence, we use a CNN for protein sequence feature extraction. First, we perform a k-mer encoding operation on the amino acid sequence. This encoding lays a solid foundation for subsequent feature extraction, thus enabling the sequence information to be processed by the network in a more suitable form. Subsequently, we carefully design a three-layer CNN block specifically for protein feature extraction. In each convolutional layer, we deploy multiple convolutional kernels. Each of these convolutional kernels, similar to a sensitive detector, focuses on learning the embedding representation of a specific region of the sequence. For each protein sequence, the convolutional kernels perform convolutional calculations in an orderly manner. Each convolutional kernel is tasked with extracting the information of a specific segment in the sequence, thus mining the potential features of the sequence from different perspectives. Inspired by the residual network, we ingeniously connect a feature aggregation module between different convolutional layers. This module is composed of a pooling layer and an activation function. We set the pooling layer as a nonlinear pooling function, specifically, the “max-pooling” operation. Under max-pooling, the length of the sequence feature vector is halved after each pooling step, effectively reducing data redundancy. The convolutional layer connected after the pooling layer is equivalent to performing a linear weighting on the result of the nonlinear function. This design not only strengthens the function of the pooling layer in reducing information redundancy but also minimizes the information loss caused by the pooling operation. Finally, we splice the result of the pooling operation with that of the convolutional operation to obtain the high-level embedding features of the protein:


(6) HT=⊕l=13(σ(W(l)X(l−1)+b(l))||MaxPool(W(l)X(l−1)+b(l)))

where ⊕ represents cross-layer vertical splicing (stacking), *‖* represents in-layer horizontal splicing, $W^{\left(l\right)}$ and $b^{\left(l\right)}$ are the learnable weight matrix (i.e. filter) and bias vector in the *l*-th layer of the CNN, respectively, and $H_{T}$ is the feature matrix corresponding to each protein sequence. These features contain rich protein sequence information and provide a solid basis for subsequent target feature representation learning and related research.

Next, we use contrastive loss to align the features of the two modalities of drugs and targets. We assume that *N* pairs of drug-target data are present and that their feature representations are $(D_{i}$, $T_{i})$, where $D_{i}=\mathrm{Concat}(H_{a},H_{m},H_{am})$. If drug *i* interacts with target *i*, we treat it as a positive sample; otherwise, as a negative sample. The contrastive loss function can be formulated as follows:


(7) Lcot=-1N∑i=1Nlog exp (t·cos ⁡(Di, Ti+))∑j∈P(i)  exp (t·cos ⁡(Di, Tj))

where $D_{i}$ and ${T_{i}}^{+}$ represent the drug feature vector and the target feature vector in the *i*-th positive sample pair, respectively, and $\cos(D_{i},\;{T_{i}}^{+})$ represents the cosine similarity between $D_{i}$ and ${T_{i}}^{+}$. t is a temperature parameter that is used to adjust the sensitivity of the loss function. *P*(*i*) represents the set of all target feature vectors paired with the i-th drug feature vector $D_{i}$, including the positive sample ${T_{i}}^{+}$ and all negative samples ${T_{i}}^{-}(j\neq i)$.

### 2.3 Single-modal feature enhancement module

In the single-modal feature enhancement module, we employ unique similarity calculation methods and loss functions to preserve the internal relationships within each modality. For the drug feature matrix and the target matrix after feature extraction, we use matrix SVD to extract the principal components, thereby effectively eliminating redundant information while accurately retaining key information. We then introduce the Gaussian kernel function to measure and calculate similarities between different drugs and between different targets in the embedded feature space. Moreover, in the original feature spaces of drugs and targets, we normalize the data, use SVD for information extraction and the Gaussian kernel function to calculate internal similarities of the data in each modality. We measure the similarity of each modality’s data in the original and embedded feature space via the consistency loss of the similarity matrix and sum the loss values of the two modalities’ data to obtain the final single-modal preservation loss. We hope that through the single-modal feature enhancement process, we can effectively avoid overlooking the complexity of the internal feature distribution of each modality due to the excessive pursuit of intermodal alignment when data resources are relatively limited. Therefore, we design the single-modal preservation loss as follows:


(8) Lpre=L cos (SD0,SD)+L cos (ST0,ST)

where $S^{0}$ and S represent the similarity matrices of single-modal data in the original and embedded feature space, respectively, $S=\text{exp}\;(-\frac{{\left||\hat{X_{i}}-\hat{X_{j}}|\right|}^{2}}{2\sigma^{2}})$, and $\hat{X_{i}}$ and $\hat{X_{j}}$ represent the features of the *i*- and *j*-th samples in the reconstructed matrix after performing SVD on cross-modal data in the feature space. $L_{\;\text{cos}\;}(\cdot )$ represents the consistency loss of the similarity matrix:


(9) L cos (S1,S2)=1N∑i=1N|1-∑j=1NS1(i,j)·S2(i,j)|

Therefore, the complete training loss of DrugDL is as follows:


(10) L=αL cos (SD0,SD)+βL cos (ST0,ST)+γLcot

where the coefficients *α*, *β*, and *γ* serve as weights to balance the importance of different loss terms and α+β+γ=1. Moreover, we output the embedding representations $D_{i}$ and $T_{i}$ of drugs and targets in the feature space, which are used to support the learning of downstream tasks.

### 2.4 Downstream drug molecule property prediction

With regard to the crucial downstream tasks of drug discovery, we constructed a multitask prediction framework that covers core scenarios such as DTI, DTA and DTBR prediction, the prediction of drug physicochemical properties, toxicity assessment, and DDI. For tasks that require cross-modal integration (e.g. DTI, DTA, and DTBR), we fuse drug and target representations into a joint feature vector through feature splicing and input it into the prediction model. For single-modal tasks (e.g. physicochemical properties and toxicity prediction), we directly use drug molecule features as inputs. To achieve efficient modeling, we adopt a hybrid framework. For structured features, in addition to training traditional machine learning models such as support vector machines (SVMs), random forests (RF), and logistic regression (LR), we also design fully connected neural networks (FCNNs) with different architectures for in-depth feature mapping. The FCNN we designed contains multiple hidden layers, uses the ReLU activation function and batch normalization to accelerate convergence, and ultimately generates prediction probabilities via a sigmoid or softmax output layer. Specific details of predictions for different downstream tasks are provided in Supplementary Tables 7–9.

During the model optimization process, we determine the optimal hyperparameters through a grid search and fivefold cross-validation. To improve prediction robustness, we adopt a hierarchical ensemble strategy: for the same task, we train both traditional machine learning models and FCNN, and fuse their prediction results on the basis of weighted voting. The weights were allocated dynamically according to the performance on the validation set. Experimental results revealed that this hybrid framework significantly improved the generalization ability of complex tasks while ensuring interpretability. For example, in binding affinity prediction, traditional models were good at capturing linearly correlated features, whereas FCNN effectively modeled nonlinear interactions of molecular embeddings. The complementarity of the two reduced the integrated prediction error. All the models were trained in a supervised learning manner, using task-specific labeled datasets for end-to-end fine-tuning. Finally, thresholding or regression outputs were used to meet the different requirements of classification and regression tasks.

### 2.5 Baselines and performance measurement

To systematically evaluate the performance of the DrugDL framework, we constructed a multilevel baseline system tailored to specific downstream tasks. This system encompasses traditional molecular fingerprints and state-of-the-art computational models (such as DrugBAN, ZeroBind, and MeTDDI). The detailed selection logic and the core characteristics of all baseline models utilized in our comparative experiments are comprehensively described in Supplementary Note 2.

In this study, we comprehensively evaluated the performance of the model in both classification and regression tasks with multiple evaluation metrics. In terms of classification tasks (e.g. DTI prediction, prediction of some drug physicochemical properties, toxicity prediction), the test results were presented and analyzed using four core metrics: accuracy (ACC), area under the receiver operating characteristic curve (AUC), specificity, and recall. For multiclassification scenarios (e.g. DDIs), to facilitate a more detailed performance analysis, we further introduced the average specificity and average recall of each category. In regression tasks (e.g. DTA prediction, drug AUC FC values, and prediction of some drug physicochemical properties), we selected the root mean square error (RMSE), coefficient of determination (R^2^), mean absolute error (MAE), and consistency index (CI) as key evaluation metrics. In addition, in some tasks (e.g. DTA prediction), to ensure a fair comparison with baseline models, we used the variant $r_{m}^{2}$ of the squared correlation coefficient value for evaluation and analysis.

## 3 Results

### 3.1 Performance of DrugDL in DTI, DTA and DTBR prediction tasks

**Figure 2:**
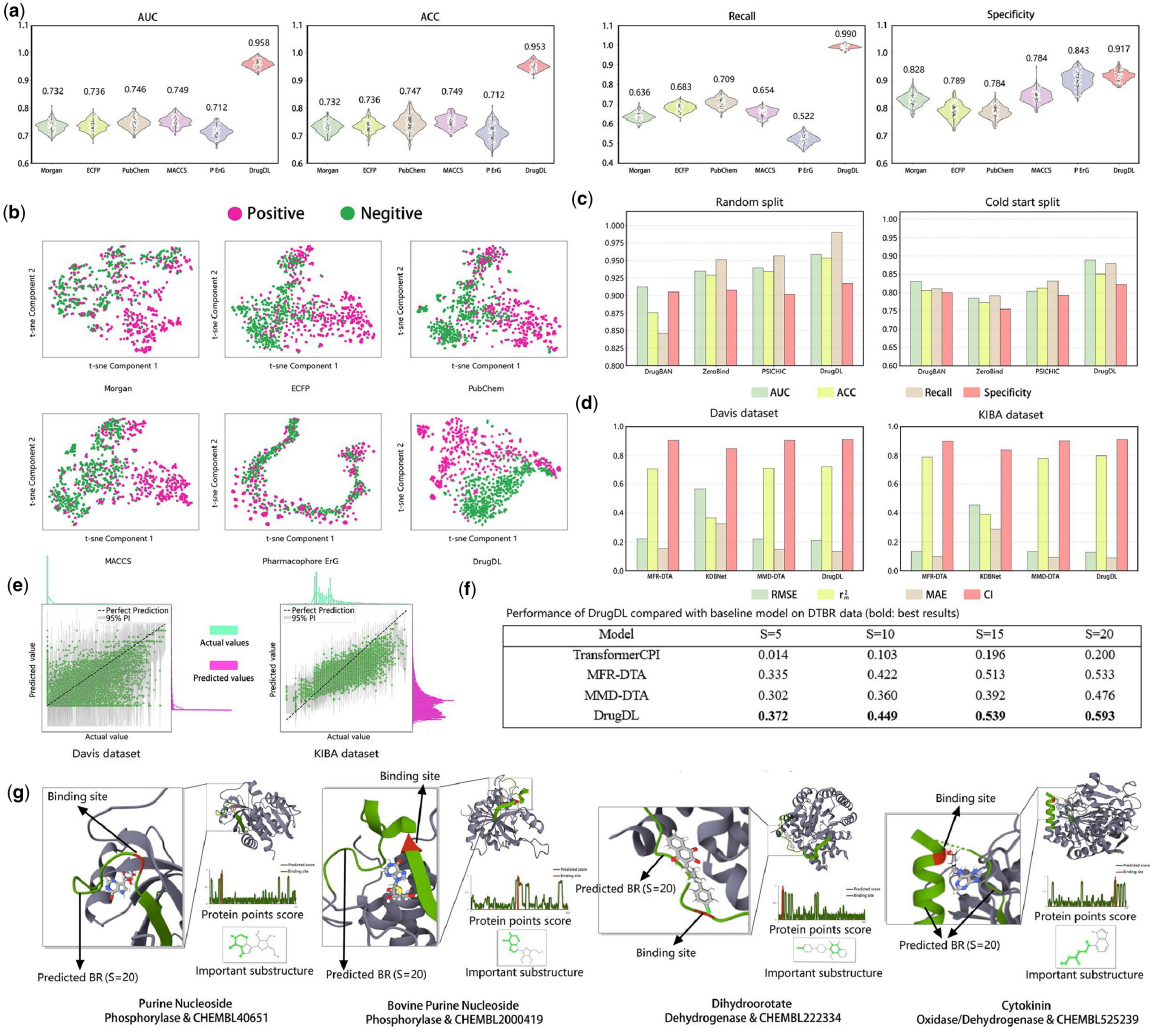
Performance evaluation of the DTI, DTA and DTBR prediction tasks. (a) Performance of DrugDL and various molecular fingerprint features was evaluated using AUC, ACC, recall, and specificity on the DTI benchmark dataset. (b) Comparison of t-SNE visualization results of various molecular fingerprint features on the DTI benchmark dataset. (c) Performance evaluation of DrugDL and baseline models in terms of the AUC, ACC, recall and specificity on randomly split and cold-start split datasets from the DTI benchmark dataset. (d) Performance evaluation of DrugDL and baseline models in terms of RMSE, $r_{m}^{2}$, MAE and CI on the Davis and KIBA DTA datasets. (e) Prediction visualization results of DrugDL on the Davis and KIBA DTA datasets. (f) Performance evaluation of DrugDL and baseline models on the DTBR dataset (sc-PDB). (g) Prediction visualization results of DrugDL on four complexes in the DTBR (sc-PDB) dataset.

The DTI prediction task is a classification task, the goal of which is to determine whether a given drug-target pair interacts. In this study, we first combined the features extracted by DrugDL with five common molecular fingerprint features: Morgan fingerprints, ECFP fingerprints, PubChem fingerprints, MACCS fingerprints, and P ErG (Pharmacophore ErG) fingerprints. For the benchmark datasets, we utilized the DTI prediction task to evaluate the performance of each feature. As shown in Fig. 2a, DrugDL consistently outperformed competing methods across all evaluation metrics on the DTI benchmark datasets. Compared with other fingerprint features, the DrugDL model exhibited significant advantages in terms of the recall and specificity metrics. We attribute these advantages primarily to the imbalance between positive and negative samples in the DTI dataset. In such cases, all fingerprint features perform better on the specificity metric than the recall metric. These findings indicate that in situations involving class imbalance, most fingerprint features may mistakenly classify many truly interacting drug-target pairs as negative samples, thereby increasing the risk of missed detections. However, the good performance of DrugDL in both recall and specificity metrics indicates that, in the DTI prediction task, the latent representations extracted by DrugDL can flexibly adapt to changes in dataset size and effectively address the sample imbalance problem, thus demonstrating strong robustness and reliability. In addition, the t-SNE visualization in Fig. 2b is consistent with these results: traditional fingerprints exhibit poor discriminability with regard to positive/negative samples, whereas DrugDL effectively separates samples into distinct clusters, demonstrating better feature discriminability and robustness.

The comparative analysis of DrugDL and the state-of-the-art DTI prediction models [DrugBAN (Bai *et al.* 2023), ZeroBind (Wang *et al.* 2023), and PSICHIC (Koh *et al.* 2024)] on both randomly split data and cold-start split data is shown in Fig. 2c. Notably, DrugDL significantly outperforms all baseline models not only on the randomly split data but also on the more challenging cold-start split data, where it exhibits excellent performance. In the cold-start evaluation, although the performance of all the models declined, DrugDL still maintained a relative advantage. Its AUC value is 0.889, which is much greater than that of DrugBAN (0.829), ZeroBind (0.784), and PSICHIC (0.803). In addition, DrugDL also performed well in ACC, recall, and specificity, with values of 0.850, 0.878, and 0.822, respectively, thereby fully demonstrating its strong ability to address cold-start problems. These results achieved by DrugDL are closely related to its various modules. Therefore, Supplementary Fig. S1 illustrates the impact of different feature extraction methods and distinct modules within DrugDL on the model’s predictive performance.

For the regression task of DTA prediction, we selected three baseline models specifically designed for this task, MFR-DTA (Hua *et al.* 2023), KDBNet (Luo *et al.* 2023), and MMD-DTA (Zhang *et al.* 2024), to facilitate an in-depth comparative analysis with DrugDL. Using Davis and KIBA datasets, we comprehensively evaluated the prediction performance of each model. As shown in Fig. 2d, DrugDL significantly outperformed all baseline models across all evaluation metrics. Its $r_{m}^{2}$ value was much higher than those of the other models, indicating a strong correlation between the predicted and actual values. DrugDL also achieved the lowest RMSE and MAE among all models, further confirming its high prediction accuracy. In addition, the concordance index (CI) of DrugDL was relatively high, underscoring the reliability of its predictions. Figure 2e intuitively shows the prediction visualization results of DrugDL on the two datasets. The points on its scatter plot are closely distributed around the diagonal, clearly indicating minimal deviation between predicted and actual values. In the KIBA dataset, the label distribution is relatively normal; that is, the actual values of the DTAs are more evenly distributed, which provides favorable conditions for the model to learn a more accurate mapping relationship.

For the more refined DTBR prediction task, we selected TransformerCPI (Chen *et al.* 2020), MFR-DTA, and MMD-DTA for performance evaluation and comparative analysis with DrugDL. To objectively assess prediction capabilities, we designed a standard evaluation by using the probability that the actual binding site falls within the predicted region as the core metric. We set varying lengths of predicted DTBRs, comprising *S* amino acids (*S* = 5, 10, 15, 20), to evaluate model performance across different segment lengths. The results, shown in Fig. 2f, present the prediction accuracy of each model under different *S* values. The data presented in the table clearly show that as *S* increases, the accuracy of the predicted binding regions of all methods improves. When *S* is equal to 5 and 10, although the prediction accuracy of all baseline models is relatively low, DrugDL exhibits significant advantages on shorter sequence segments. When *S* is 15 or 20, the prediction performance of DrugDL is further enhanced, with accuracies reaching 0.539 and 0.593, respectively. Compared with the baseline models, the prediction performance of DrugDL is far superior when *S* = 15 and *S* = 20, providing further strong verification for its effectiveness and reliability in the DTBR prediction task. Figure 2g illustrates the alignment between DrugDL-predicted binding regions (*S* = 20, green) and experimentally verified actual binding sites (red) for the four drug-target pairs. For three proteins, the predicted binding regions accurately encompass the actual sites. For cytokinin oxidase/dehydrogenase, the predicted site is slightly offset but nevertheless within the predicted region. Additionally, we visualized drug molecules based on motif features, in which red areas identify key substructures in drug–protein binding. Collectively, these results underscore the strong predictive ability of DrugDL, which is capable not only of predicting drug-target interactions and binding affinities but also of identifying specific binding regions.

### 3.2 DrugDL for molecular property assessment and toxicity profiling

**Figure 3:**
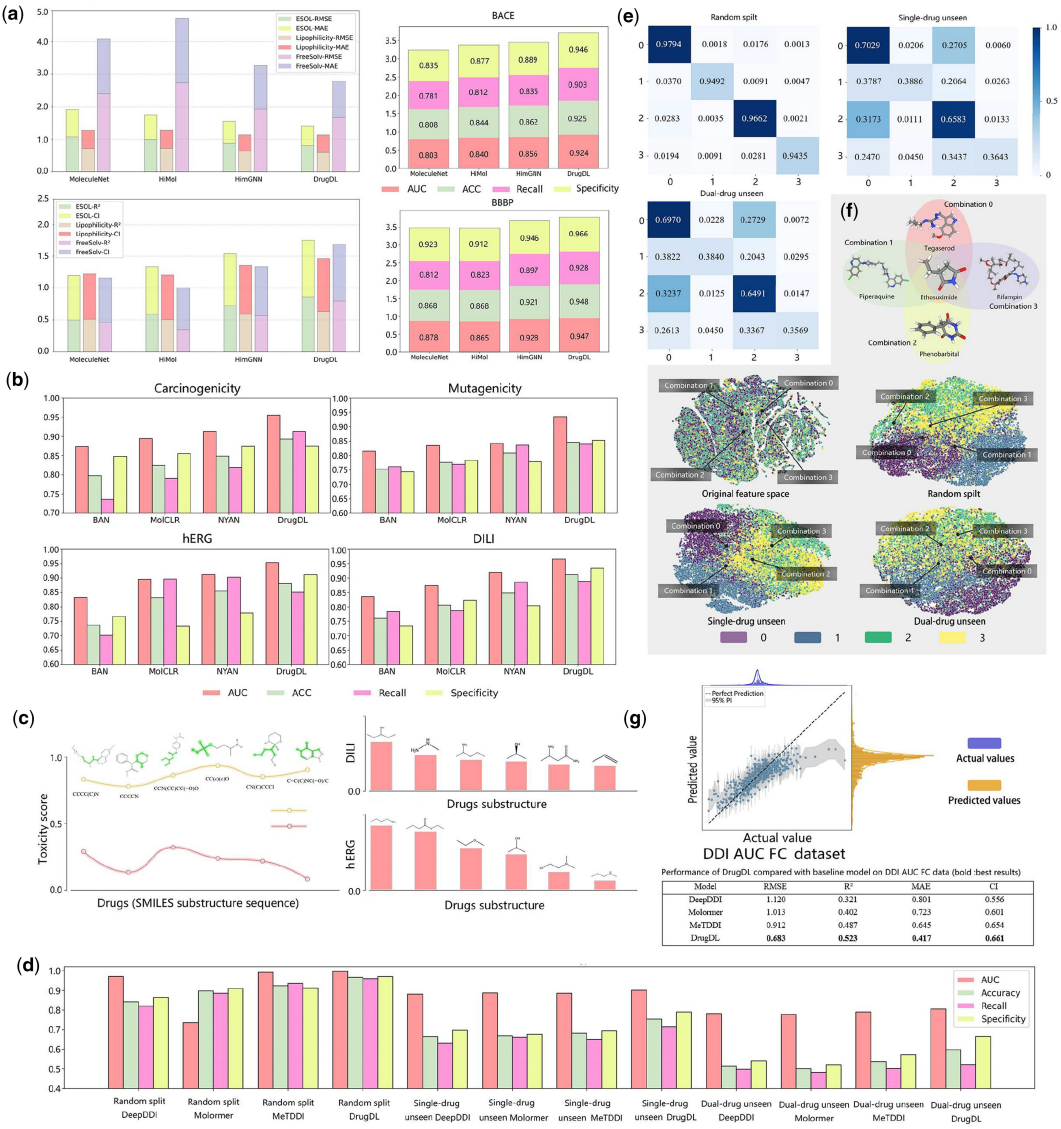
Performance evaluation of the drug safety prediction task. (a) Performance evaluation of DrugDL and baseline models on datasets of the physicochemical properties of drug molecules (BACE, BBBP, ESOL, Lipophilicity, and FreeSolv). (b) Performance evaluation of DrugDL and baseline models in terms of the AUC, ACC, recall and specificity on datasets of the toxicity of drug molecules (carcinogenicity, mutagenicity, DILI and hERG). (c) Comparison of toxicity before and after the removal of substructures identified by DrugDL and analysis of sensitive areas. (d) Performance evaluation of DrugDL and each baseline model in terms of the AUC, ACC, recall and specificity for the DDI dataset in three scenarios: a randomly split dataset, a single-drug unseen dataset and a dual-drug unseen dataset. (e) Classification confusion matrices of DrugDL in three DDI dataset segmentation scenarios. (f) Prediction visualization results of DrugDL in the three DDI dataset segmentation scenarios, using Ethosuximide, Tegaserod, Piperaquine, Phenobarbital and Rifampin as examples. (g) Performance comparison between DrugDL and baseline models on the DDI AUC FC value dataset, along with prediction visualization results.

We first evaluated the performance of DrugDL on five benchmark datasets representing the physicochemical properties of drug molecules: BACE, BBBP, ESOL, Lipophilicity, and FreeSolv. The prediction results of each model are shown in Fig. 3a. Overall, DrugDL exhibited better performance than other baseline models in all evaluation metrics. For the BACE dataset, the AUC, ACC, recall, and specificity of DrugDL were 0.923, 0.924, 0.903, and 0.946, respectively, which were higher than those of the other models. Similarly, on the BBBP dataset, DrugDL also reached the highest values for these four indicators. Although the prediction effects of HiMol (Zang *et al.* 2023) and MoleculeNet were similar and HimGNN (Han *et al.* 2023) was slightly better than the former two in terms of AUC and ACC, their performances were inferior to those of DrugDL. On the ESOL, Lipophilicity, and FreeSolv datasets for regression tasks, DrugDL also performed very well.

For drug toxicity prediction, we compared DrugDL with three advanced baseline models across four key toxicity datasets: carcinogenicity, mutagenicity, hERG cardiotoxicity, and drug-induced liver injury. As shown in Fig. 3b, DrugDL significantly outperformed all baseline models in terms of the AUC, ACC, and specificity metrics, demonstrating strong predictive capability and stability. These findings highlight the advantages of DrugDL with respect to its ability to capture drug molecule toxicity features. On the DILI dataset, while the recall of DrugDL was slightly lower than those associated with NYAN and MolCLR, these baseline models achieved higher recall values by classifying most data as positive, which increased false positives and reduced both specificity and overall ACC. In contrast, DrugDL effectively balanced recall and specificity, reducing false positives and enhancing model reliability and practicality. Thus, DrugDL offers greater clinical value in terms of its comprehensive performance, particularly in minimizing unnecessary diagnoses and misjudgments. Additionally, we used DrugDL’s substructure extraction method to identify substructure sequences crucial for toxicity judgment and presented them in Supplementary Table S1, including substructure images, toxicity, and SMILES representations. When these substructures were removed and toxicity tests were conducted, the results (Fig. 3c) confirmed that removal of the substructures identified by the model reduced the toxicity of the compounds. Figure 3c also highlights the six substructures most sensitive to different toxicities, providing valuable insights into the drug molecule toxicity mechanisms.

### 3.3 DrugDL accurately predicts DDI and pharmacokinetic impact

We evaluated DrugDL on DDI prediction tasks by comparing it with three baseline models: DeepDDI (Ryu *et al.* 2018), Molormer (Zhang *et al.* 2022), and MeTDDI (Zhong *et al.* 2024). The performance results on the DDI benchmark dataset are shown in Fig. 3d. First, in the randomly split dataset, DrugDL demonstrated excellent performance. In all evaluation metrics, DrugDL significantly outperformed all the baseline models. However, when we divided the dataset into single-drug unseen and dual-drug unseen subsets, the prediction performance of all models decreased significantly. Nevertheless, DrugDL maintained excellent prediction performance in both scenarios. The classification results for the three datasets are shown in Fig. 3e, and the confusion matrices correspond to the results in Fig. 3d. Figure 3f further shows the t-SNE visualization results of DrugDL in the three classification tasks and the original feature space. We focused on the drug ethosuximide to represent its four relationships with other drugs (i.e. Tegaserod, Piperaquine, Phenobarbital, and Rifampin). From the t-SNE visualization results, we observed that the DrugDL model successfully separated the four types of data in the randomly split dataset, with well-defined boundaries between various types of data. For the unseen datasets, although the prediction results for these data were not as good as for the randomly split data, the data points retained a similar clustering trend. These findings further demonstrate the advantages of the DrugDL model in mitigating the cold-start problem.

To evaluate pharmacokinetic impact, we assessed predictions of the area under the plasma concentration-time curve fold-change (AUC FC) values. As shown in Fig. 3g, the prediction performance of DrugDL on the DDI AUC FC value data was better than that of the baseline models. The scatter plot clearly indicates that the distribution of the predicted values is approximately the same as that of the actual values, and the 2D points composed of the actual and predicted values are basically distributed on the diagonal. This result not only proves the effectiveness and accuracy of the DrugDL model but also provides strong support for its application in fields such as drug R&D and drug interaction evaluation.

### 3.4 DrugDL identifies potential SARS-CoV-2 and metabolic enzyme inhibitors

To explore the practical effectiveness of DrugDL in real-world drug development and clinical applications in depth, we systematically compared its performance with that of other baseline models [MolCLR (Wang *et al.* 2022), NYAN (Lam *et al.* 2023), and ImageMol (Zeng *et al.* 2022)] in two key areas: anti-SARS-CoV-2 drug development and metabolic enzyme inhibitor identification, thereby verifying its potential in real-world application scenarios.

**Figure 4:**
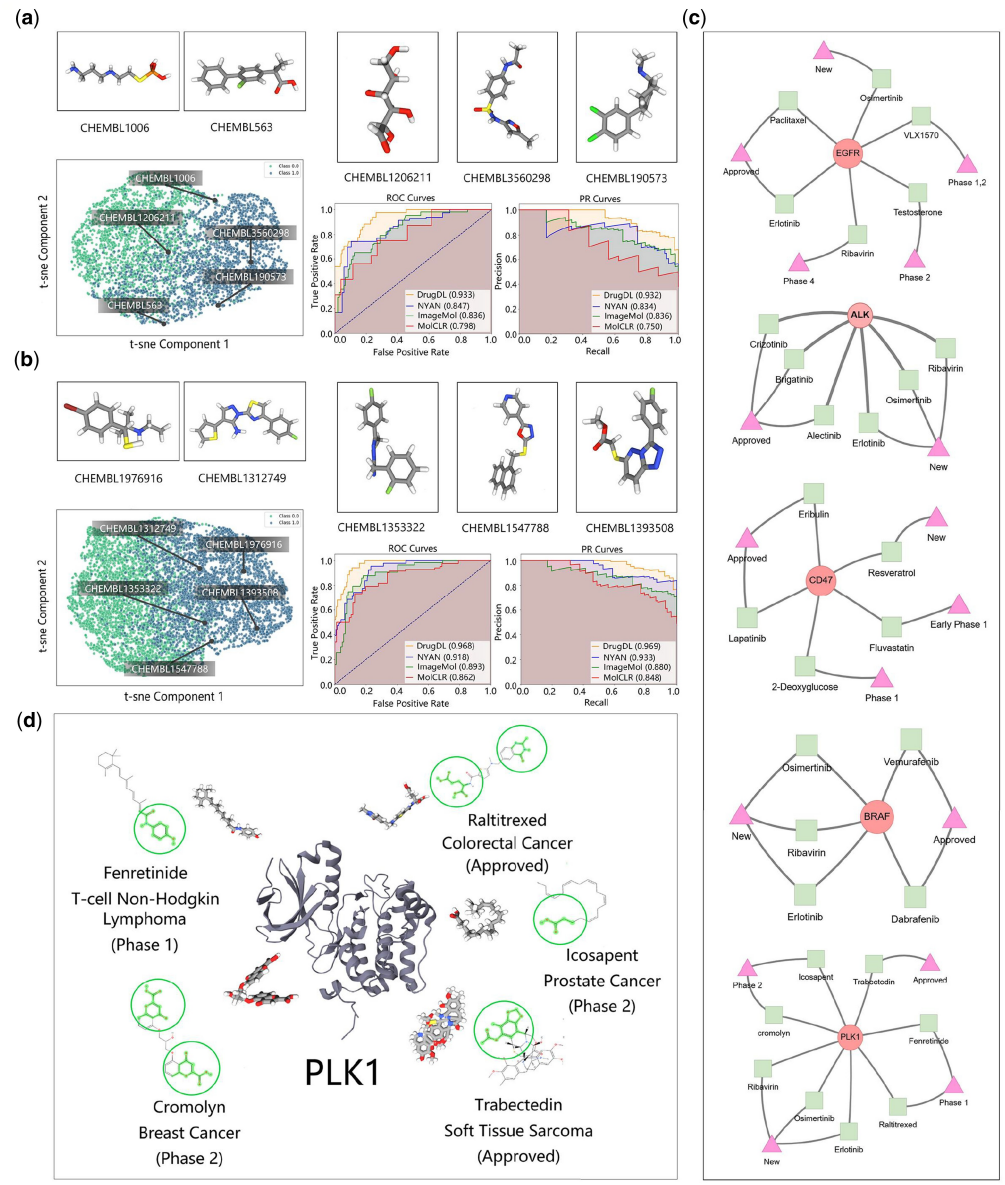
Evaluation of practical efficacy in real-world drug R&D and clinical applications. (a) Performance evaluation of DrugDL and baseline models on the SARS-CoV-2 inhibitor dataset. (b) Performance evaluation of DrugDL and baseline models on the metabolic enzyme (CYP2C9) inhibitor dataset. (c) Analysis of DrugDL in the prediction of potential drugs for tumor targets (BRAF, ALK, EGFR, CD47, and PLK1). (d) Visualization of drug molecules predicted by DrugDL to interact with the PLK1 target.

In anti-SARS-CoV-2 drug development, SARS-CoV-2 is the causative virus of the COVID-19 pandemic. During the life cycle of SARS-CoV-2, the 3CL protease is a crucial part of virus replication (Zhou *et al.* 2020). We evaluated the antiviral potential of drugs by predicting and analyzing whether drug molecules can bind to the 3CL protease and inhibit its activity. The relevant results (Fig. 4a) show that in terms of the AUC and AUPR, DrugDL demonstrated significant advantages over all baseline models, achieving scores of 0.933 and 0.932, respectively. Moreover, features extracted by DrugDL achieved clear clustering of drug molecules according to their anti-SARS-CoV-2 activity.

In metabolic enzyme inhibitor identification, the CYP2C9 metabolic enzyme, a representative metabolic enzyme, plays an important role in the metabolism of many commonly used drugs in the clinic (McKinney *et al.* 2009). Through the prediction and analysis of whether a drug is a CYP2C9 inhibitor, DrugDL also demonstrated excellent performance. According to the ROC curve evaluation (Fig. 4b), the AUC value of DrugDL reached 0.968, which was significantly greater than those associated with the other baseline models. In PR curve evaluation, the AUPR value of DrugDL was also 0.969, which means that DrugDL maintained an effective balance between high precision and recall even in the case of unbalanced samples. In conclusion, the cross-field application potential of DrugDL has been demonstrated in two different fields: anti-SARS-CoV-2 drug development and CYP2C9 enzyme inhibitor identification.

### 3.5 The predictive identification of cancer therapeutics and mechanistic analysis via DrugDL

To further evaluate the reliability of DrugDL in practical application scenarios, we selected multiple tumor-related targets for in-depth case studies. These targets included BRAF (B-Raf proto-oncogene) (Kusano *et al.* 2010), ALK (anaplastic lymphoma kinase) (Voena *et al.* 2025), EGFR (epidermal growth factor receptor) (Ciardiello and Tortora 2008), CD47 (a key transmembrane protein) (Guo *et al.* 2023), and PLK1 (an important protein in cell cycle regulation) (Raab *et al.* 2018). For each target, DrugDL was first pre-trained on a benchmark dataset and subsequently used to predict potential drug-target interactions.

As shown in Fig. 4c, we present several drugs with high predicted interaction probabilities for each target. For the BRAF target, two drugs, Vemurafenib and Dabrafenib, have been approved to treat melanoma and other related cancers, which initially validated the effectiveness of our prediction method (Chapman *et al.* 2011). Although no direct clinical trial evidence is yet available, our results suggest that Erlotinib and Osimertinib may exhibit significant interactions with BRAF. Notably, these two drugs have exhibited potential anticancer activities in multiple studies and have been supported by clinical trials. These findings indicate that the candidate drugs predicted by DrugDL to interact with specific targets are promising candidates for further exploration in cancer treatment. In the prediction of PLK1 targets, Fenretinide is in a phase 1 clinical trial for T-cell non-Hodgkin lymphoma (Makena *et al.* 2021), and Cromolyn and Icosapent are in phase 2 clinical trials for breast cancer and prostate cancer (Peterson *et al.* 2021). For the CD47 target, the predicted drug Fluvastatin has entered an early phase 1 clinical trial for malignant melanoma (Zhang *et al.* 2011).

In addition, we analyzed the DTI mechanisms of five drug molecules (i.e. Fenretinide, Cromolyn, Trabectedin, Icosapent, and Raltitrexed) that bind to the PLK1 target. Figure 4d depicts the 3D structures of these molecules, as well as the attention weights of the corresponding atoms and motifs. We highlighted the substructures with large attention weights in green. These highlighted sections help analyze and understand in depth the complex and subtle drug-target interactions, thus enhancing our understanding of the key functional groups in drug molecules.

### 3.6 Experimental validation of EGFR- and ALK-targeting drug candidates and toxicity assessment

**Figure 5:**
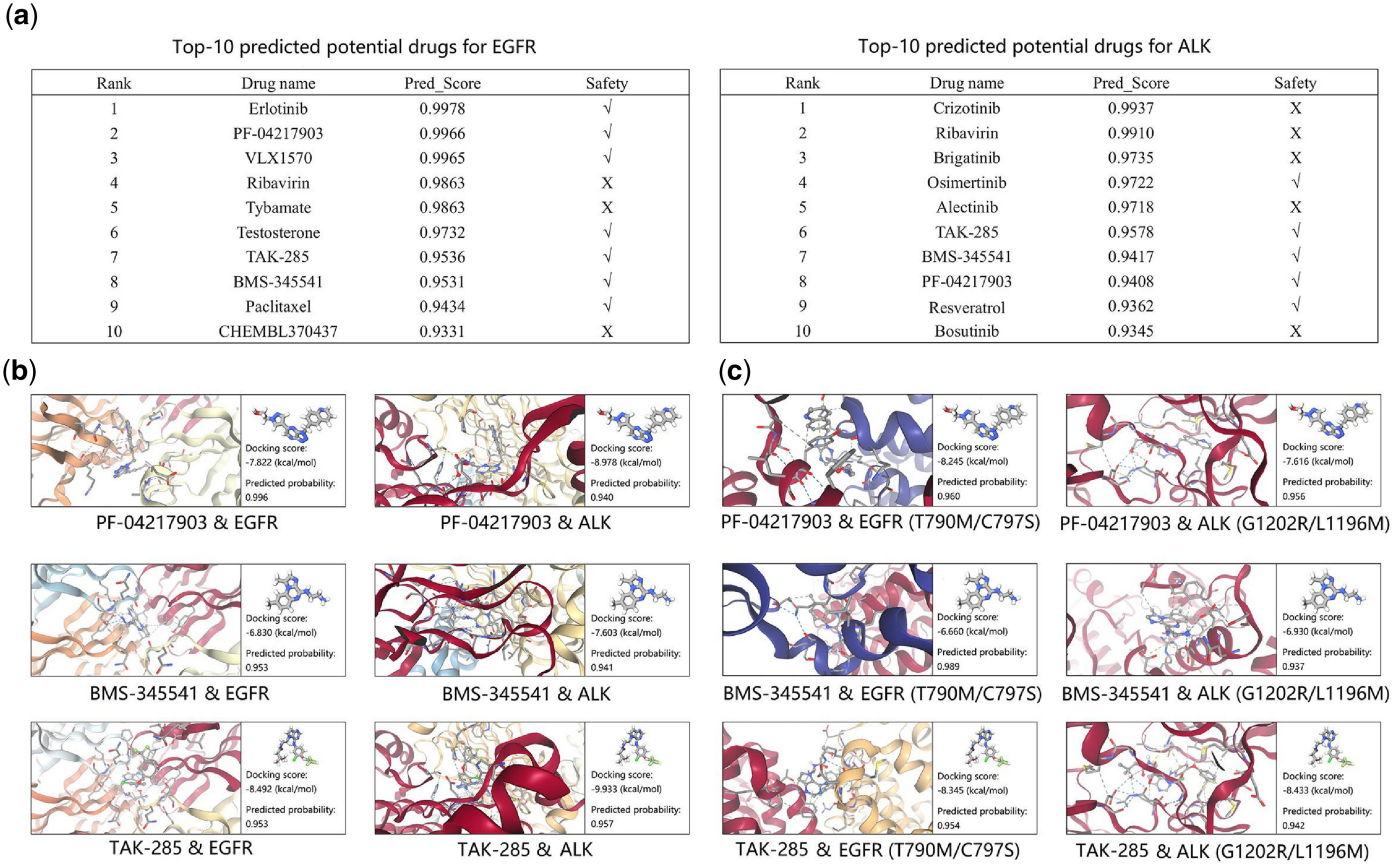
Experimental verification and toxicity evaluation results of EGFR- and ALK-targeted drug candidates identified by DrugDL. (a) Results of DrugDL predictions for the top 10 potential drugs binding to the EGFR target and the top 10 potential drugs binding to the ALK target. (b) Docking poses and scores of the predicted interactions between three potential dual-target drugs (PF-04217903, BMS-345541, and TAK-285) and the wild-type EGFR and ALK targets. (c) Docking poses and scores of the predicted interactions between the three potential dual-target drugs and EGFR and ALK mutants.

To assess virtual drug screening, we evaluated DrugDL-identified candidates for EGFR and ALK targets. Figure 5a shows the top 10 predicted drugs for each wild-type protein. DrugDL identified 7 of the 10 EGFR inhibitors and 5 of the 10 ALK inhibitors as safe. Among the safe EGFR inhibitors, Erlotinib, VLX1570, Testosterone, and Paclitaxel have been externally validated. PF-04217903, BMS-345541, and TAK-285 were predicted to be safe and to bind both EGFR and ALK. Further validation was conducted by performing molecular docking experiments with AutoDock Vina (Trott and Olson 2010).

Molecular docking analysis (Fig. 5b) revealed that the binding free energies (Δ*G*) of the three candidate drugs with the wild-type EGFR and ALK ranged from −6.8 to −10.0 kcal/mol. Figure 5c shows the docking poses and scores of the three candidate drugs against EGFR and ALK mutants. According to thermodynamic criteria (Δ*G* < 0), these values indicate that the binding processes between the drug molecules and target proteins are spontaneous and exhibit moderate to strong affinity. Notably, PF-04217903 and TAK-285 showed particularly strong affinities for ALK (Δ*G* < −8.9 kcal/mol), while TAK-285 also bound well to EGFR (Δ*G* = −8.492 kcal/mol), further highlighting the relative advantages of these drugs in terms of binding to specific targets.

In clinical practice, tumor cells often undergo genetic mutations, leading to alterations in the structure and function of target proteins. In recent years, EGFR mutations such as T790M and C797S have emerged as resistance-causing mutations for third-generation drugs. The T790M mutation alters the spatial structure of the ATP-binding pocket in EGFR, impeding drug-target binding, whereas the C797S mutation may disrupt covalent binding between drugs and EGFR, rendering the drugs ineffective. Similarly, ALK mutations such as G1202R and L1196M can alter the protein structure, making it difficult for drugs to bind to the mutated ALK protein and reducing their antitumor activity. Consequently, identifying novel drug candidates capable of addressing target mutations represents an urgent need.

Thus, we predicted the interaction scores of the three dual-target drugs with the two mutated targets using DrugDL. The results indicated that all three drugs were capable of interacting with both target proteins. Docking experiments further validated these predictions, showing that the Δ*G* values of the three candidate drugs against EGFR and ALK mutants ranged from –6.6 to –8.5 kcal/mol, indicative of strong binding affinities. Additionally, the docking results between the candidate drugs and other mutated EGFR and ALK proteins are presented in Supplementary Table S3.

## 4 Discussion and conclusion

We present DrugDL, a cross-modal deep learning framework for drug molecule representation and multi-property prediction. This framework integrates multilevel drug and target protein data through multichannel GNNs and CNNs, captures multiscale interactions via cross-modal learning and single-modal enhancement, and preserves feature attributes to minimize information loss. Our experiments show that DrugDL outperforms existing methods in predicting DTIs, DTAs, DTBRs, drug physicochemical properties, toxicity, and DDIs. Moreover, DrugDL identifies crucial molecular substructures with pharmacological insights, thereby assisting drug researchers in screening key action sites and uncovering local interaction mechanisms. Its practical utility has been demonstrated in real-world R&D and clinical applications, providing theoretical support for drugs at various clinical trial stages by predicting cancer-target interactions and experimentally validating the efficacy and safety of EGFR/ALK-targeted candidates.

Although DrugDL’s core paradigm involves representing drug molecule features and predicting downstream properties by analyzing DTIs, the availability of such interaction data is not the primary bottleneck limiting its performance. In particular, the single-modal feature enhancement module in DrugDL independently models drug and target modalities, thereby preserving their distinct representations and preventing information loss during multimodal fusion. This module facilitates indirect inference of key information via intramodal information transfer and cross-modal collaborative learning, even under limited direct DTI data, by explicitly capturing correlations within modal data (e.g. drug–drug and target-target similarities). Compared with prior methods, DrugDL is less constrained by data availability, an advantage that was fully validated by evaluations in both cold and warm start scenarios. We explicitly mitigate gradient conflicts across the diverse classification, regression, and binding site prediction tasks by employing hierarchical ensemble learning with weighted voting, task-specific fully connected neural network branches, and separate loss optimization for each downstream task, which decouples conflicting gradients during joint training and ensures stable multi-task prediction without performance degradation.

In deep learning-based biological discovery, where prediction mechanisms often remain black-box in nature (Shen *et al.* 2025), DrugDL excels in parsing pharmacologically informative substructures. By converting drug molecules into graph representations via a pharmacophore-motif-guided substructure splitting approach, DrugDL extracts multiscale pharmacology-related substructures (e.g. pharmacophores and toxicophores). Through attention mechanisms and interpretability techniques, it quantifies each substructure’s contribution to pharmacological indicators and generates hypotheses with mechanistic insights. Substructure analysis serves as an “interpretability anchor” for deep learning models, partially elucidating their prediction logic and guiding virtual screening and molecular design optimization to accelerate drug discovery. The successful application of DrugDL in EGFR/ALK-targeted drug discovery further validates its capacity for effective feature extraction and decision-making. However, despite the interpretability potential of this approach in drug property prediction, most model outputs still require wet-lab experimental validation. Future researchers may employ advanced technologies to systematically validate the biological credibility of DrugDL-identified substructures, with the aim of enhancing drug structure optimization and simultaneously improving both drug safety and efficacy.

## Supplementary Material

btag392_Supplementary_Data
