# Supplementary material for: DrugDL: dual-modal deep learning framework for multi-property drug prediction and targeted therapy discovery
Source: Bioinformatics. 2026 Jun 15;42(7):btag392. doi: 10.1093/bioinformatics/btag392 (PMC13332442; doi:10.1093/bioinformatics/btag392)
Supplement: btag392_Supplementary_Data [file btag392_supplementary_data.docx]

**Supplementary Information**

**DrugDL: Dual-modal deep learning framework for multi-property drug prediction and targeted therapy discovery**

Qi Zhang^1, #^, Xuan Yu^2, #^, Yuxiao Wei^3, #^, Yunpeng Xia^4^, Long-Chen Shen^1^, Zhi-Hui Wang^1^,

Hong-Bin Shen^4^, and Dong-Jun Yu^1, *^

**Author Information**

Authors and Affiliations

**^1^ School of Computer Science and Engineering, Nanjing University of Science and Technology, 200 Xiaolingwei, Nanjing, 210094, China**

Qi Zhang, Zhi-Hui Wang, Long-Chen Shen & Dong-Jun Yu

**^2^ Department of Computer Science, City University of Hong Kong, 83 Tat Chee Avenue, Kowloon Tong,**

**Hong Kong, 999077, China**

Xuan Yu

**^3^ School of Computer Science & Technology, Beijing Jiaotong University, Beijing, 100044, China**

Yuxiao Wei

**^4^ Institute of Image Processing and Pattern Recognition, Shanghai Jiao Tong University, 800 Dong Chuan Rd., Minhang District, Shanghai 200240, China**

Yunpeng Xia, Hong-Bin Shen

^#^ These three authors contributed equally to this work.

**Corresponding authors**

Correspondence should be addressed to: Dong-Jun Yu, [njyudj@njust.edu.cn](mailto:njyudj@njust.edu.cn)


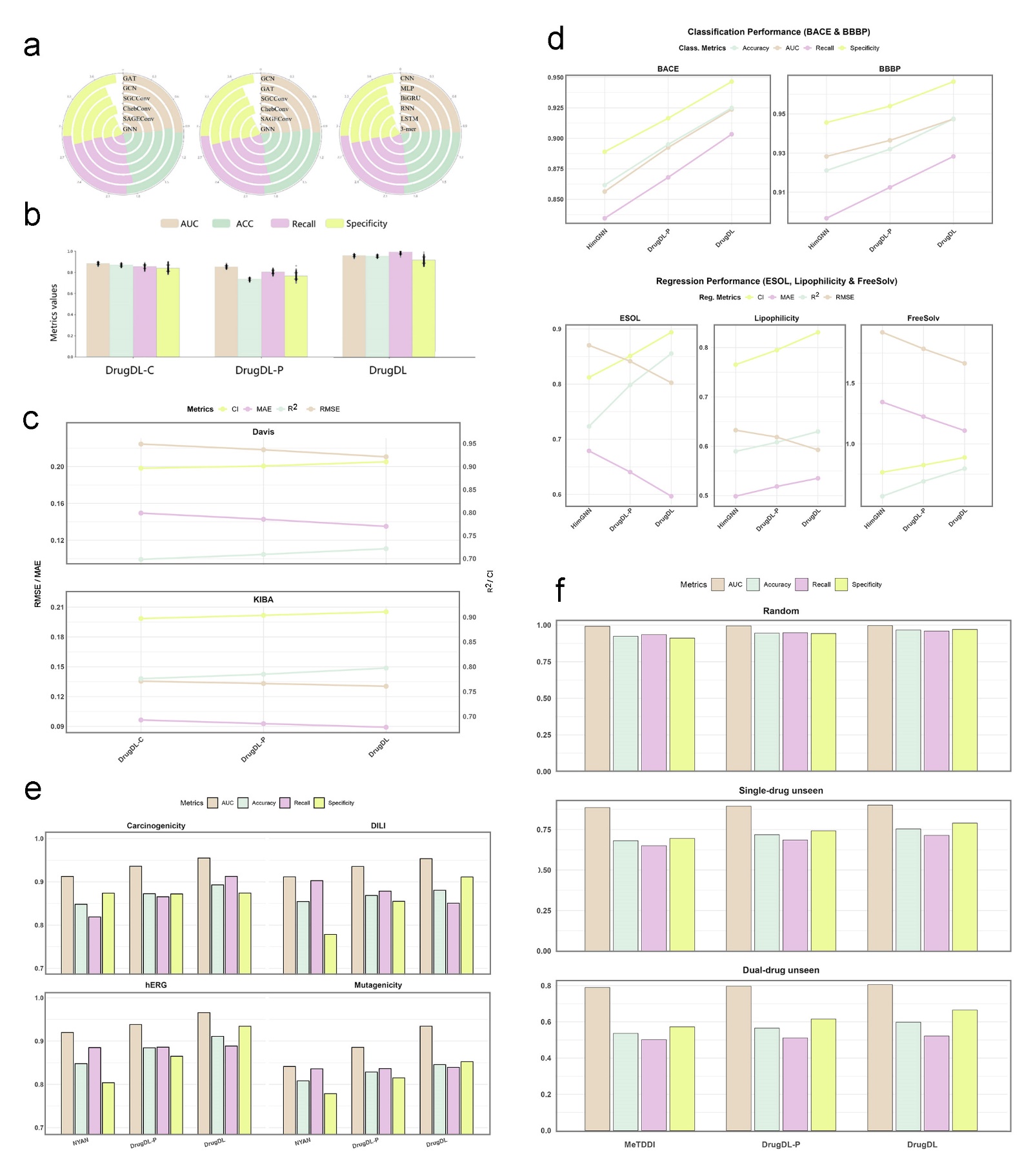


**Supplementary Figure 1** Analysis results of ablation experiments. **a**, Performance evaluation of different GNNs and target feature extraction methods in the DrugDL model on the DTI dataset. **b**, Evaluation of the impact of the cross-modal interaction learning module (DrugDL-C) and the single-modal feature enhancement module (DrugDL-P) in the DrugDL model on the model's performance on the DTI dataset. **c**, Performance comparison of model variants on DTA prediction using Davis and KIBA datasets. **d**, Ablation results on drug physicochemical property prediction tasks (BACE, BBBP, ESOL, Lipophilicity, and FreeSolv). **e**, Ablation results on molecular toxicity prediction tasks (Carcinogenicity, DILI, hERG, and Mutagenicity). **f**, Ablation results on DDI prediction under three different data split scenarios (Random, Single-drug unseen, and Dual-drug unseen).


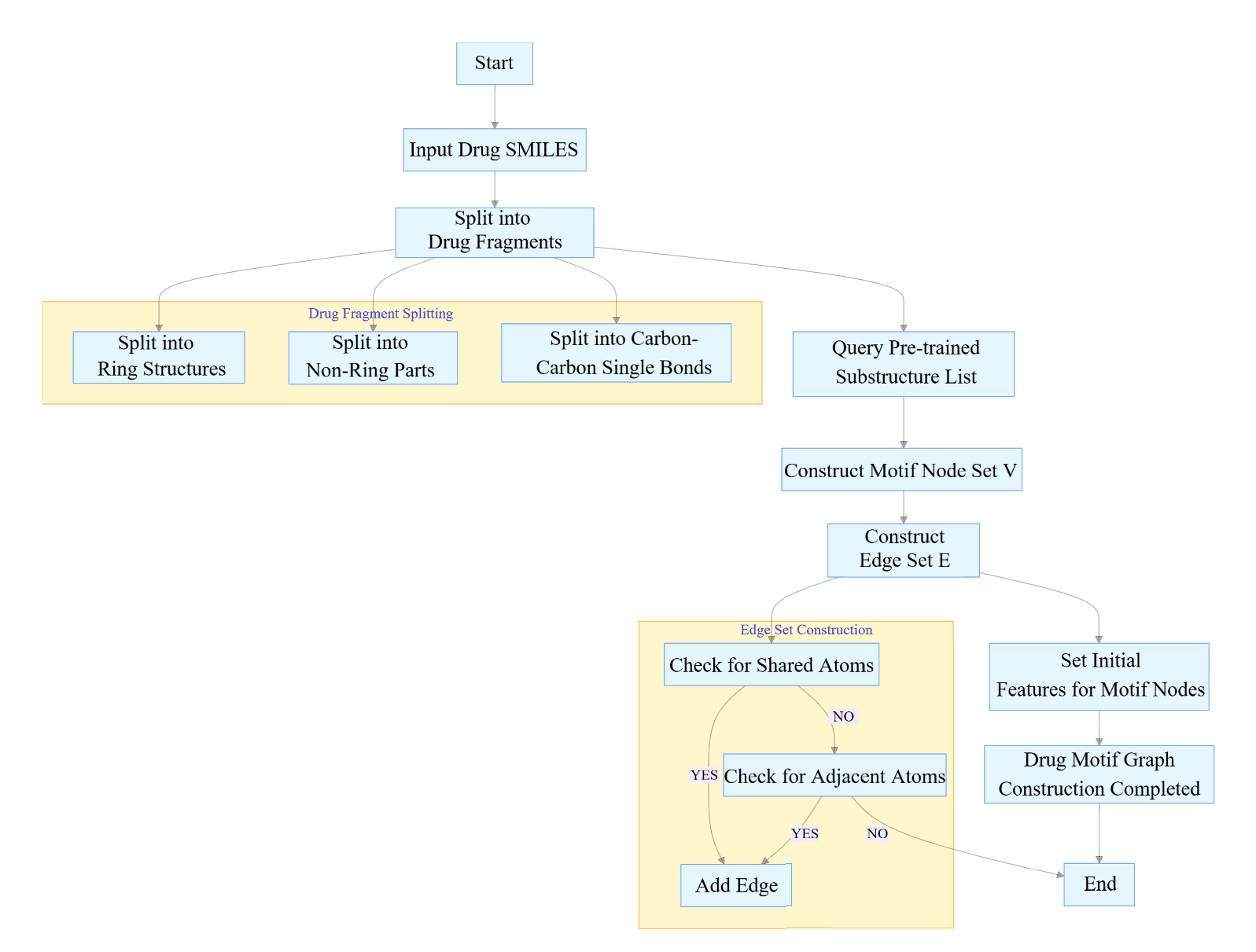
**Supplementary Figure 2** Drug motif graph construction flowchart. This flowchart illustrates the step-by-step process of constructing a drug motif graph, which involves splitting a drug molecule into various fragments, constructing motif nodes and edges, and setting initial features for the motif nodes. The graph is built based on the rules of shared atoms and adjacent atoms between motifs, providing a powerful tool for revealing complex interactions and functional relationships between drug molecules.

**
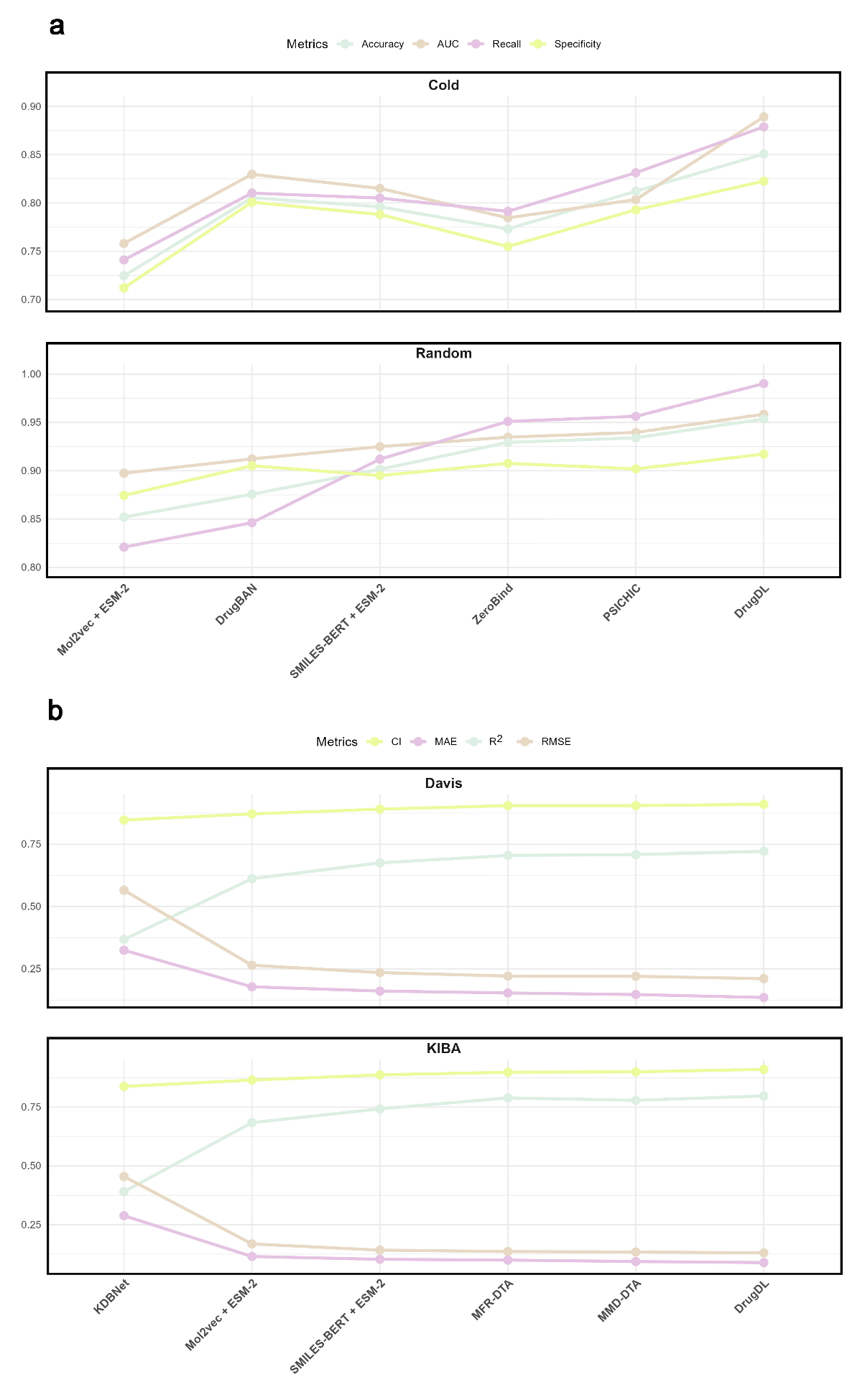
**

**Supplementary Figure 3** Performance comparison with mainstream pre-trained models on DTI and DTA tasks. **a**, DTI prediction performance comparison under random and cold split settings, including Mol2vec + ESM-2, DrugBAN, SMILES-BERT + ESM-2, ZeroBind, PSICHIC, and DrugDL. Metrics include AUC, Accuracy, Recall, and Specificity. **b**, DTA prediction performance comparison on Davis and KIBA datasets, including KDBNet, Mol2vec + ESM-2, SMILES-BERT + ESM-2, MFR-DTA, MMD-DTA, and DrugDL.

**Supplementary Table 1** Molecular substructures identified by DrugDL that play a significant role in drug toxicity.

| **Toxicity** | **Substructure** | **SMILES** |
| --- | --- | --- |
| Carcinogenicity |  | C=C(c)c1ccccc1CCc |
| Carcinogenicity |  | CN(C)[C@@H](Cc(cc)cc)C(N)=O |
| Carcinogenicity |  | CC(=O)NCC(=O)N(C)C |
| Mutagenicity |  | C=C/C=C/CCC |
| DILI |  | Cc1ccsc1C |
| Mutagenicity |  | cc(c)C(c1ccccc1)C(Cl)ClS |
| DILI |  | Cc1cccc(-c)c1 |
| Carcinogenicity |  | CC[C@@H](CO)NC(C)=O |
| Mutagenicity |  | CC(C)O[C@@H]1O[C@@H](C)C[C@H](O)[C@H]1O |
| DILI |  | cc(c)C(CC)c1ccccn1 |
| Mutagenicity |  | CNCC(=O)NC(C)C |
| Carcinogenicity |  | cC(N)=O |
| DILI |  | cNc1ccc(C)cc1CC(=O)O |
| Carcinogenicity |  | NC(=O)[C@@H](N)CS |
| Carcinogenicity |  | CN/C(NN)=C1\C=CC=CC1=O |
| Mutagenicity |  | ccc(cc)-c1ccnc(c)n1nc |
| Carcinogenicity |  | CCCn1nc(C)n(C)c1=O |
| Mutagenicity |  | CNN |
| Mutagenicity |  | cn(c)-c1ccc(Cl)cc1C |
| Mutagenicity |  | ccc(c(c)=O)c(n)n |
| Carcinogenicity |  | C/C=C/C(C)O |
| Carcinogenicity |  | N[C@@H]1C[C@H](N)[C@@H](O)C[C@H]1O |
| hERG |  | cc(c)CNC |
| hERG |  | C=C(c)c1ccccc1CCc |
| hERG |  | CN/C(NN)=C1\C=CC=CC1=O |
| Mutagenicity |  | CC(=O)NCC(=O)N(C)C |
| Mutagenicity |  | CC(N)C(=O)N(C)C |
| DILI |  | CC(C)=N |
| Carcinogenicity |  | cC(O)CCCN(C)c |
| Carcinogenicity |  | CCCNC |
| Carcinogenicity |  | O=[N+]([O-])O |
| hERG |  | cNC(C)=O |
| Carcinogenicity |  | CCN(CC)CC(=O)O |
| hERG |  | CCC[C@@H](C)N |
| DILI |  | CNC(=O)C(C)N |
| Mutagenicity |  | CCCn1nc(C)n(C)c1=O |
| DILI |  | CCc1cnc[nH]1 |
| DILI |  | CC(N)=O |
| Carcinogenicity |  | cc1cccc(OC)c1 |
| DILI |  | COc1ccccc1O |
| Carcinogenicity |  | C[C@H](C)O |
| Mutagenicity |  | CCC(OC)C(N)=O |
| Carcinogenicity |  | ccc(CC)c(c)C |
| hERG |  | CCCCN |
| hERG |  | CC(N)CC(N)=O |
| hERG |  | cc(c)C1(CC)C(=O)NC(=O)NC1=O |
| Mutagenicity |  | cc(c)C(=O)c1cc(I)cc(I)c1 |
| DILI |  | CCNC(C)c |
| Mutagenicity |  | Cc1ccccc1N(c)C |
| Carcinogenicity |  | CC(C)(c)[C@@H](Oc)C(C)=O |
| hERG |  | CCN(CC)CC(=O)O |
| DILI |  | CN/C(NN)=C1\C=CC=CC1=O |
| Mutagenicity |  | Cc1cccc2c1CCO2 |
| Carcinogenicity |  | CC(c)(c)O |
| hERG |  | CC(N)C(=O)N(C)C |
| hERG |  | cc1nc(c)c(c(c)C)oc-1c(c)C |
| DILI |  | CCCN(C)C |
| Carcinogenicity |  | cc(c)[C@H](CC)c1ccccn1 |
| hERG |  | cN(C)C(N(C)C)C(C)(C)c |
| DILI |  | CCCC(=O)OCC |
| Carcinogenicity |  | cC(O)c1ccccc1 |
| hERG |  | C=C1N(CC)c2cccc(c)c2C1(C)C |
| Mutagenicity |  | CCN[C@H](C)/C(C)=N/O |
| DILI |  | C=C(c)c1ccccc1CCc |
| DILI |  | Cc1cccc2c1CCO2 |
| Carcinogenicity |  | CC(N)C(=O)N(C)C |
| hERG |  | C=C/C=C/CCC |
| hERG |  | cc1ccc(OC)c(OC)c1 |
| Mutagenicity |  | COc1ccc(C)c(Cl)c1Cl |
| Carcinogenicity |  | cc1cNc2ccccc2C1CC |
| DILI |  | CCC(O)C(C(=O)O)C(O)CC |
| Carcinogenicity |  | CC(N)CC(C)O |
| hERG |  | cC([N+])Cc1cccc(O)c1 |
| DILI |  | C=C(O)NCc |
| hERG |  | ccc(c(c)O)C(C)C |
| Carcinogenicity |  | CCc1ncc[nH]1 |
| Mutagenicity |  | NCC(=O)N[C@@H](CS)C(N)=O |
| Mutagenicity |  | NC1CC(N)C(O)CC1O |
| Mutagenicity |  | Cc1cccc2c1CCO2 |
| hERG |  | C=C/C=C/CCC |
| DILI |  | CC=CC(C)(C)C |
| hERG |  | C[C@H](C)O |
| DILI |  | CCCC(C)N |
| DILI |  | CCCCN |
| hERG |  | COCCO |
| DILI |  | CCC(CC)NC |
| hERG |  | CC(=O)NC(C)C |
| hERG |  | C[C@@H]1CC=CCN1CC |
| Mutagenicity |  | C=C(C)NC(=O)/C(C)=C\C |
| DILI |  | CCN(C(C)=S)c1ccccc1C(c)=N |
| hERG |  | ccc(cc)-c1ccnc(c)n1nc |
| DILI |  | C[C@H](N)C(=O)N(C)C |
| hERG |  | CC(C)C(O)C(C)N |
| Mutagenicity |  | CC(C)C |
| hERG |  | cc1c(n)ncn1C |
| Mutagenicity |  | COCC(C)(C)N |
| DILI |  | CCOC |
| Mutagenicity |  | NC(=O)C(N)CS |
| DILI |  | CC(=O)NCC(=O)N(C)C |
| Mutagenicity |  | CN(C)CCCCn |

**Supplementary Table 2** Evaluation of the performance of DrugDL and baseline models in terms of ACC, Recall and Specificity on SARS-CoV-2 inhibitor (3CL) dataset and metabolic enzymes (CYP2C9) inhibitor dataset.

| Dataset | Model | ACC | Recall | Specificity |
| --- | --- | --- | --- | --- |
| CYP2C9 | MolCLR | 0.8723 | 0.8501 | 0.8512 |
|  | ImageMol | 0.8956 | 0.8834 | 0.8923 |
|  | NYAN | 0.9047 | 0.8921 | 0.8912 |
|  | DrugDL | **0.9286** | **0.9111** | **0.9487** |
| 3CL | MolCLR | 0.8823 | 0.8910 | 0.8750 |
|  | ImageMol | 0.9001 | 0.9087 | 0.8525 |
|  | NYAN | 0.9112 | 0.9198 | 0.8835 |
|  | DrugDL | **0.9474** | **0.9652** | **0.9489** |

**Supplementary Table 3** The docking poses and scores of the predicted interactions between three potential dual-target drugs (PF-04217903, BMS-345541, and TAK-285) and the EGFR (T790M) and ALK (L1198F) targets.

| Drug | Target | Mutation | Molecular docking | Docking score (kcal/mol) | Predicted probability |
| --- | --- | --- | --- | --- | --- |
| PF-04217903 | EGFR | T790M | 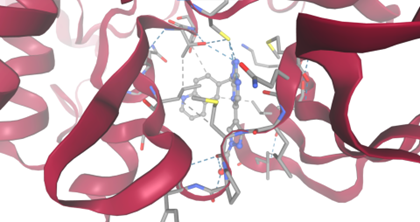 | -7.342 | 0.9785 |
| BMS-345541 | EGFR | T790M | 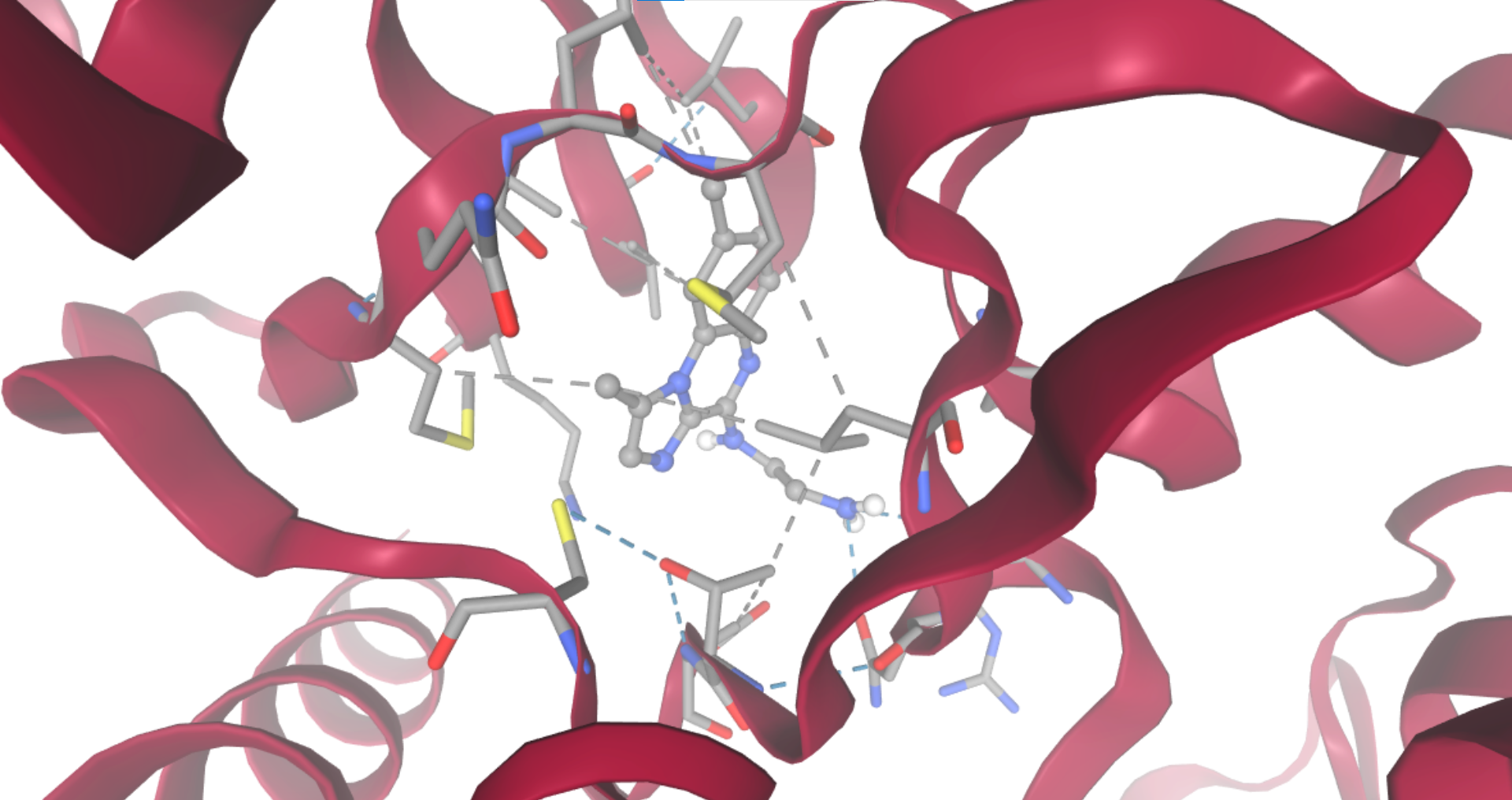 | -6.403 | 0.9546 |
| TAK-285 | EGFR | T790M | 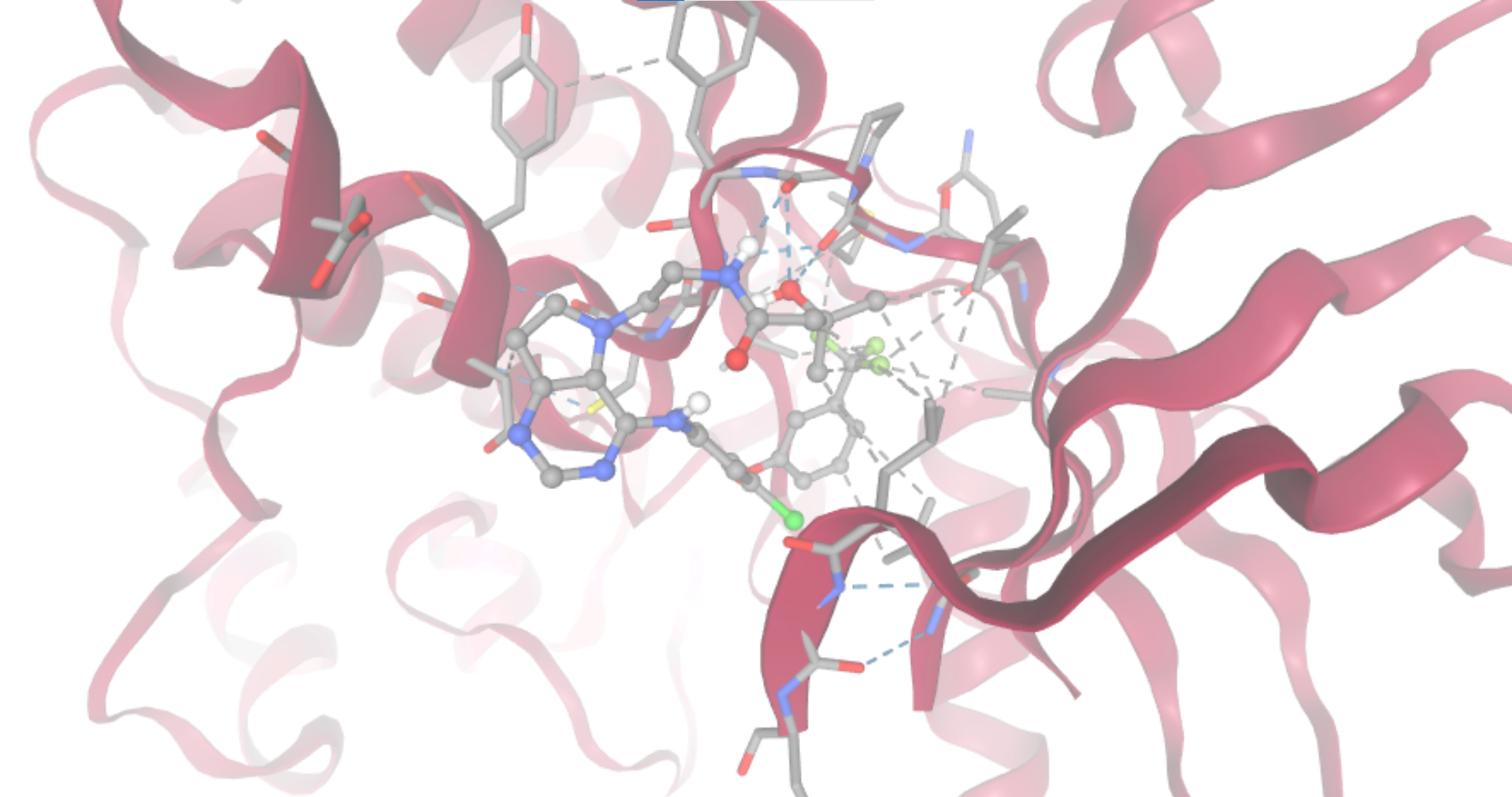 | -7.571 | 0.9687 |
| PF-04217903 | ALK | L1198F | 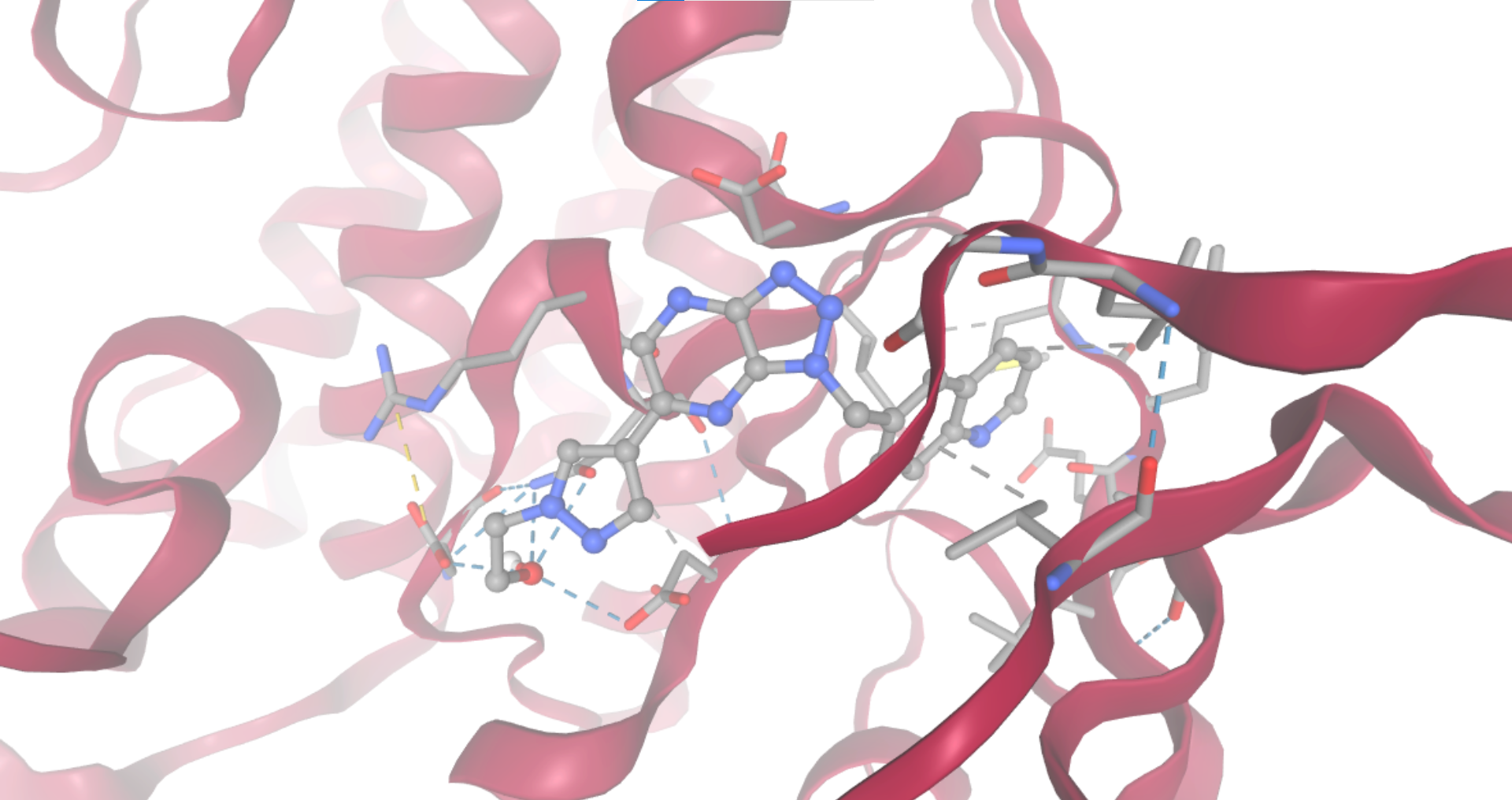 | -7.243 | 0.9772 |
| BMS-345541 | ALK | L1198F | 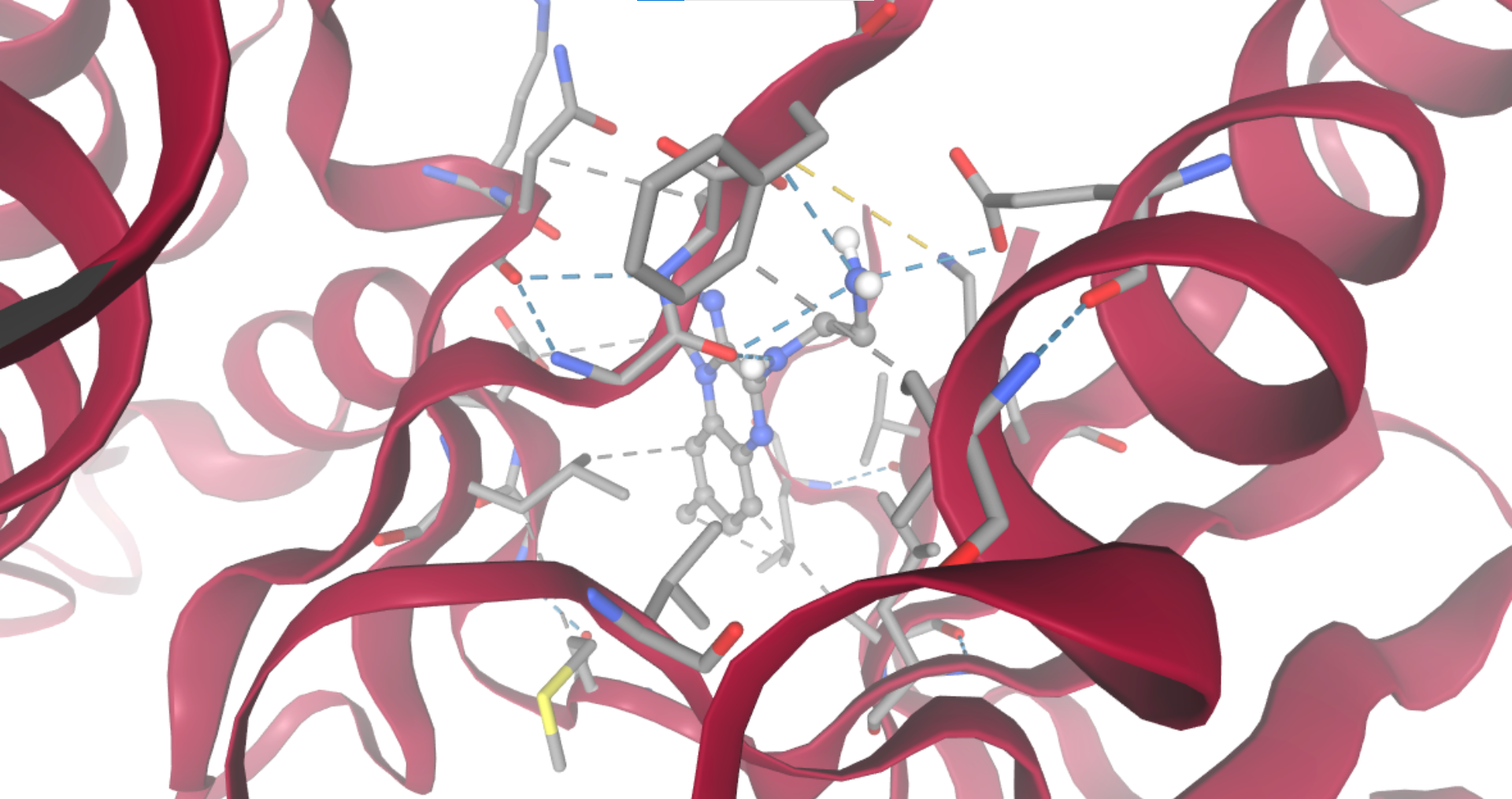 | -6.472 | 0.9621 |
| TAK-285 | ALK | L1198F | 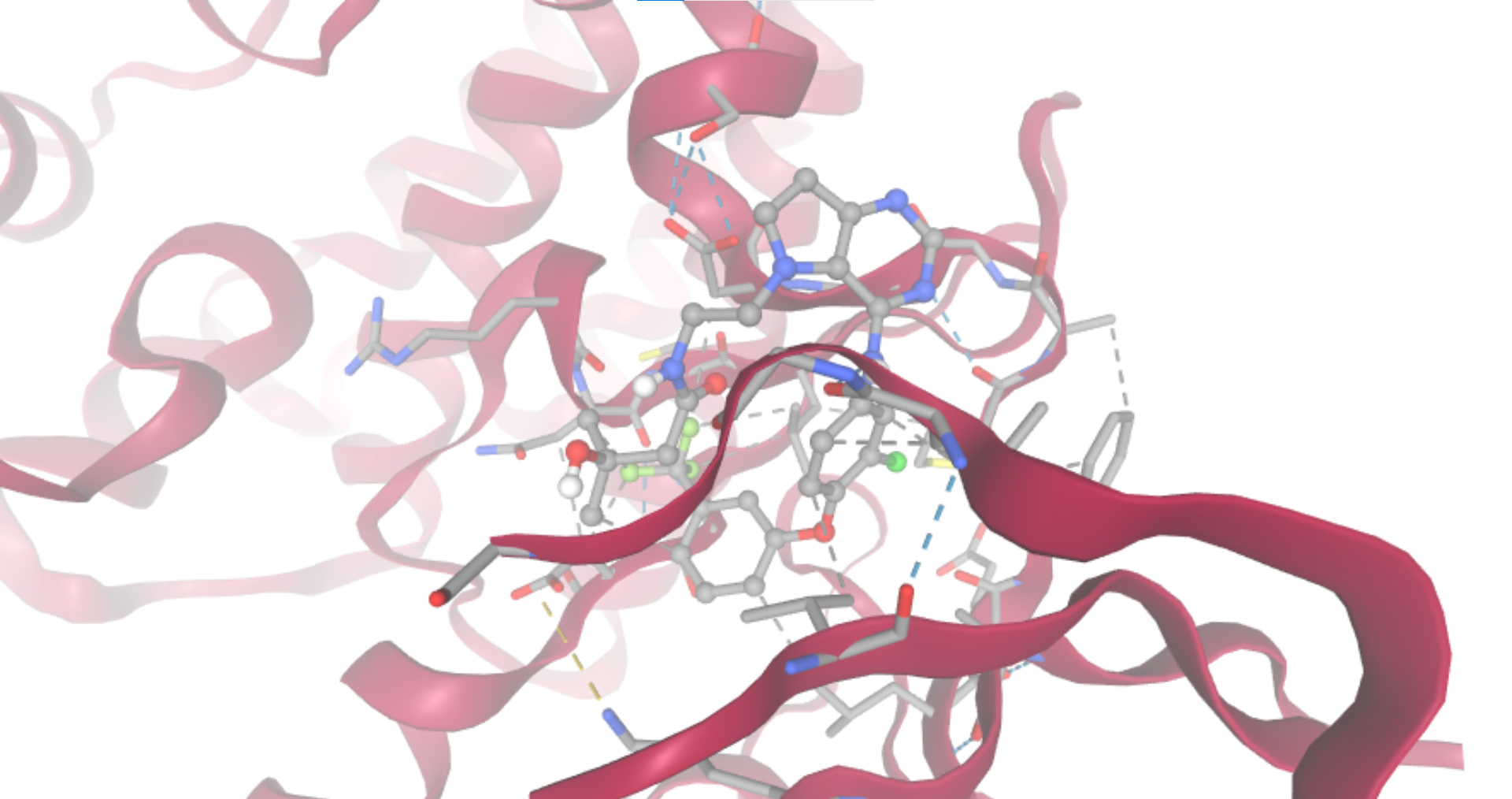 | -7.697 | 0.9898 |

**Supplementary Table 4** Experimental dataset statistics.

| Scenarios | Dataset | Drugs | Proteins | Interactions |
| --- | --- | --- | --- | --- |
| DTI | BindingDB | 14,643 | 2,623 | 49,199 |
|  | BioSNAP | 4,510 | 2,181 | 27,464 |
|  | Human | 2,726 | 2,001 | 6,728 |
| DTA | Davis | 68 | 442 | 30,056 |
|  | KIBA | 2,111 | 229 | 118,254 |
| Drug properties | BBBP | 2,039 | - | - |
|  | BACE | 1,513 | - | - |
|  | ESOL | 1,118 | - | - |
|  | FreeSolv | 636 | - | - |
|  | Lipophilicity | 4,067 | - | - |
| Drug toxicity | Carcinogenicity | 2,964 | - | - |
|  | Mutagenicity | 1.251 | - | - |
|  | hERG | 19,047 | - | - |
|  | DILI | 4,710 | - | - |
| DDI | Classification | 1,409 | - | 22,922 |
|  | Regression | 2,111 | - | 3,464 |
| Clinical applications | 3CL | 344 | - | - |
|  | CYP2C9 | 11,722 | - | - |

**Supplementary Table 5** Drug atom representation.

| Number | Feature | Dimension |
| --- | --- | --- |
| 1 | One-hot encoding of the atom element | 44 |
| 2 | One-hot encoding of the degree of the atom in the molecule, which is the number of directly-bonded neighbors (atoms) | 11 |
| 3 | One-hot encoding of the total number of H bound to the atom | 11 |
| 4 | One-hot encoding of the number of implicit H bound to the atom | 11 |
| 5 | Whether the atom is aromatic | 1 |
| All |  | 78 |

**Supplementary Table 6** Hyper parameter settings.

| Model | Hyperparameter | value |
| --- | --- | --- |
| Optimizer | Learning rate | 0.00001 |
| Mini-batch | Train batch size | 64 |
|  | Test batch size | 64 |
| Training | Number of epochs | 100 |
|  | Log interval | 10 |
| CNN for protein encoder | Number of filters | [128, 128, 128] |
|  | Kernel size | [3, 6, 9] |
| GNN for drug encoder | Input features | 78 |
|  | Hidden node dimensions | [512, 256] |
|  | Attention heads | 4 |
|  | Dropout rate | 0.2 |
| Similarity calculation | Sigma | 1.0 |
|  | SVD dimension | 50 |
| Fully connected decoder | Number of hidden neurons | 512 |
| Discriminator | Number of hidden neurons | 256 |

**Supplementary Table 7** Specific settings of predictors for DTI and DDI prediction.

| Model | Hyperparameter | value |
| --- | --- | --- |
| RF | Number of decision trees | 100 |
| LR | Solver | liblinear |
|  | Maximum number of iterations | 1000 |
| SVM | Kernel function type | Poly |
|  | Regularization parameter | 1.0 |
|  | Degree of polynomial kernel | 50 |
|  | Kernel coefficient scaling method | Scale |
| Fully connected decoder | Number of hidden layers | 3 |
|  | Hidden layer dimensions | [1024, 512, 256] |
|  | Dropout | 0.2 |
|  | Output layer dimension | 1/4 |
|  | Output layer activation function | Sigmoid/Softmax |
|  | Optimizer | Adam |
|  | Learning rate | 1e-4 |
|  | Batch size | 128 |
|  | Number of training epochs | 150 |

**Supplementary Table 8** Specific settings of predictors for drug physicochemical properties and toxicities prediction.

| Model | Hyperparameter | value |
| --- | --- | --- |
| RF | Number of decision trees | 80 |
| LR | Solver | liblinear |
|  | Maximum number of iterations | 1000 |
| SVM | Kernel function type | Poly |
|  | Regularization parameter | 1.0 |
|  | Degree of polynomial kernel | 30 |
|  | Kernel coefficient scaling method | Scale |
| Fully connected decoder | Number of hidden layers | 2 |
|  | Hidden layer dimensions | [512, 256] |
|  | Dropout | 0.2 |
|  | Output layer dimension | 1 |
|  | Output layer activation function | Sigmoid |
|  | Optimizer | Adam |
|  | Learning rate | 1e-4 |
|  | Batch size | 128 |
|  | Number of training epochs | 50 |

**Supplementary Table 9** Specific settings of predictors for DTA and DDI (AUC FU) prediction.

| Model | Hyperparameter | value |
| --- | --- | --- |
| RF | Number of decision trees | 50 |
|  | Solver | 10 |
|  | Maximum number of iterations | 6 |
| LR | Kernel function type | liblinear |
|  | Regularization parameter | 1000 |
| SVM | Degree of polynomial kernel | Rbf |
|  | Kernel coefficient scaling method | 1.0 |
|  | Number of hidden layers | 30 |
|  | Hidden layer dimensions | Scale |
| Fully connected decoder | Dropout | 0.3 |
|  | Output layer dimension | [1024, 512, 256] |
|  | Output layer activation function | 0.2 |
|  | Optimizer | 1 |
|  | Learning rate | 1e-4 |
|  | Batch size | 128 |
|  | Number of training epochs | 50 |

**Supplementary Note 1.** Core Technology Stack and Dependent Libraries.

To ensure full reproducibility of our study and facilitate the application of the DrugDL framework by other researchers, we specify the core technology stack and dependent libraries used in our experimental environment. The DrugDL model was developed and trained using the PyTorch (v2.4.1) deep learning framework, accelerated by NVIDIA RTX 5090 and 4090 GPUs. For molecular data processing and feature extraction, RDKit (v2024.03.5) was employed to parse SMILES strings and construct molecular structures, while NumPy (v1.24.3) was utilized for extensive numerical computations and matrix operations. Visual analysis and performance evaluation charts were generated using Matplotlib (v3.7.5). Furthermore, external tools and databases were integrated into our workflow: AutoDock Vina (accessed via SwissDock, https://www.swissdock.ch) was used to predict docking scores for drug-target interactions, and RCSB PDB (https://www.rcsb.org/) was utilized to retrieve and visualize interaction regions. The complete source code, pre-trained models, and comprehensive environment configuration instructions are publicly available in our GitHub repository (<https://github.com/ZhangQi99/DrugDL>).

**Supplementary Note 2.** Baseline Selection Logic and Model Characteristics.

To systematically evaluate the performance of the DrugDL framework, we constructed a multilevel baseline system from the perspective of task adaptability.

- Drug Molecule Representation: We compared traditional molecular fingerprint methods to verify the distinctiveness and information density of the features generated by DrugDL. The baselines include Morgan fingerprints (based on topological encoding of molecular substructures), ECFP (extended connectivity fingerprints), PubChem fingerprints (functional group descriptors), MACCS fingerprints (predefined key substructures), and P ErG fingerprints (spatial feature encoding of pharmacophores).
- DTI Prediction: Three types of cutting-edge models were selected: DrugBAN captures local interaction patterns between drugs and targets through a bilinear attention network; ZeroBind achieves cold-start prediction based on the prior knowledge of protein structures; and PSICHIC integrates multisource biological networks to construct a heterogeneous graph attention model.
- DTA Prediction: We compared MFR-DTA (multiscale feature recombination for optimizing binding free energy modeling), KDBNet (a two-tower architecture enhanced by knowledge distillation), and MMD-DTA (multimodal disentangled representation learning).
- DTBR Prediction: TransformerCPI (self-attention-driven residue localization), MFR-DTA (extending affinity prediction to spatial site recognition), and MMD-DTA (a cross-modal feature alignment framework) were introduced to verify the representation ability of DrugDL for microscopic binding mechanisms.
- Physicochemical Properties Prediction: MoleculeNet (a standardized multitask learning framework), HiMol (a hierarchical GNN), and HimGNN (a heterogeneous information fusion model) were used as baselines to evaluate the generalization ability of DrugDL features in tasks such as solubility and lipophilicity.
- Toxicity Prediction: BAN (bidirectional attention modeling of toxic fragments), MolCLR (contrastive learning to enhance molecular representation), and NYAN (a network for inferring toxic metabolic pathways) were compared, covering four scenarios: carcinogenicity, mutagenicity, hERG cardiotoxicity, and liver injury.
- DDI Prediction: DeepDDI (substructure interaction modeling), Molormer (a transformer-based molecular pair encoder), and MeTDDI (a multiview pharmacokinetic impact prediction framework) were selected. Model robustness was verified by predicting the relationships between drug metabolism inhibition/enhancement and the AUC FC value.

All baseline models were strictly reproduced according to the experimental settings described in their original papers to ensure fair and rigorous comparisons.

**Supplementary Note 3.** Experimental Settings and Dataset Splitting Strategies.

DrugDL and the baseline methods discussed above were evaluated on the basis of specific sample processing strategies and validation settings. To address the issue of sample imbalance, we implemented targeted treatment measures. We oversampled the positive samples and undersampled the negative samples simultaneously, thus ensuring that the number of positive samples reached a consistent level after oversampling, with the goals of balancing the distributions of positive and negative samples and providing a more balanced data foundation for subsequent model training. We subsequently divided the dataset according to strict standards and carried out experiments on this basis. Specifically, 80% of the positive and negative sample pairs were extracted as the training set, while the remaining 20% were reserved as the test set to evaluate model performance.

In the process of dividing the DTI dataset, we adopted two methods: random splitting and cold start splitting^30^. Random splitting indicates that the dataset is randomly divided into a training set and a test set according to a preset ratio, whereas cold start splitting ensures that drugs and proteins in the test set do not appear in the training set while maintaining the splitting ratio, thus preventing the model from relying excessively on known features. This strategy enables the model to exhibit a more realistic prediction ability when encountering test data rather than inferring from the learned features of drugs and proteins.

In the research field of DDIs, we followed the preprocessing standards of MeTDDI, and reversed the order of drug pairs with labels 1 and 2, thus obtaining the information of drug pairs with labels 3 and 4. Specifically, label 1 indicates that when drug $d_{1}$ and drug $d_{2}$ are used in combination, the metabolism of drug $d_{1}$ is reduced; label 2 represents that it is increased. Labels 3 and 4 represent the reduction and increase in the metabolism of drug $d_{2}$ when drug $d_{1}$ and drug $d_{2}$ are used in combination, respectively. In splitting the DDI dataset, we not only applied random splitting but also introduced more detailed cold start splitting strategies for single-drugs unseen and dual-drugs unseen. These settings enabled the model to predict interactions involving individual drugs or drug pairs not encountered during training, thereby allowing a more comprehensive evaluation of its generalization ability.
